# Insights into the molecular mechanism of ParAB*S* system in chromosome partition by *Hp*ParA and *Hp*ParB

**DOI:** 10.1093/nar/gkae450

**Published:** 2024-06-06

**Authors:** Chen-Hsi Chu, Che-Ting Wu, Min-Guan Lin, Cheng-Yi Yen, Yi-Zhan Wu, Chwan-Deng Hsiao, Yuh-Ju Sun

**Affiliations:** Institute of Bioinformatics and Structural Biology, National Tsing Hua University, Hsinchu 300, Taiwan; Institute of Bioinformatics and Structural Biology, National Tsing Hua University, Hsinchu 300, Taiwan; Institute of Molecular Biology, Academia Sinica, Taipei 115, Taiwan; Institute of Molecular Biology, Academia Sinica, Taipei 115, Taiwan; Institute of Bioinformatics and Structural Biology, National Tsing Hua University, Hsinchu 300, Taiwan; Institute of Molecular Biology, Academia Sinica, Taipei 115, Taiwan; Institute of Bioinformatics and Structural Biology, National Tsing Hua University, Hsinchu 300, Taiwan

## Abstract

The ParAB*S* system, composed of ParA (an ATPase), ParB (a DNA binding protein), and *parS* (a centromere-like DNA), regulates bacterial chromosome partition. The ParB-*parS* partition complex interacts with the nucleoid-bound ParA to form the nucleoid-adaptor complex (NAC). In *Helicobacter pylori*, ParA and ParB homologs are encoded as *Hp*Soj and *Hp*Spo0J (*Hp*ParA and *Hp*ParB), respectively. We determined the crystal structures of the ATP hydrolysis deficient mutant, *Hp*ParAD41A, and the *Hp*ParAD41A-DNA complex. We assayed the CTPase activity of *Hp*ParB and identified two potential DNA binding modes of *Hp*ParB regulated by CTP, one is the specific DNA binding by the DNA binding domain and the other is the non-specific DNA binding through the *C*-terminal domain under the regulation of CTP. We observed an interaction between *Hp*ParAD41A and the *N*-terminus fragment of *Hp*ParB (residue 1–10, *Hp*ParBN10) and determined the crystal structure of the ternary complex, *Hp*ParAD41A-DNA-*Hp*ParBN10 complex which mimics the NAC formation. *Hp*ParBN10 binds near the *Hp*ParAD41A dimer interface and is clamped by flexible loops, L23 and L34, through a specific cation-π interaction between Arg9 of *Hp*ParBN10 and Phe52 of *Hp*ParAD41A. We propose a molecular mechanism model of the ParAB*S* system providing insight into chromosome partition in bacteria.

## Graphical Abstract

**Graphical Abstract:**
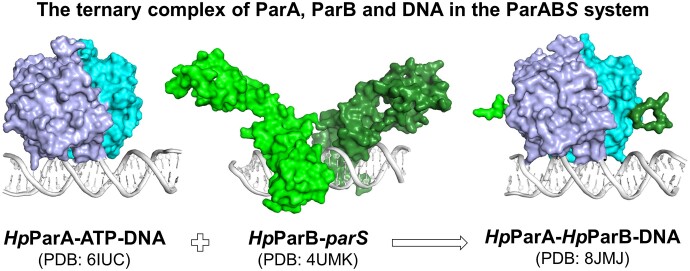

## Introduction

Accurately delivering the replicated chromosomal and plasmid DNA to each daughter cell, referred to as DNA segregation or partition, is crucial for the stable inheritance of genetic material (1). The ParAB*S* system (partitioning (*par*)) is a highly-conserved machinery responsible for DNA segregation in bacteria (2,3). This system comprises three key components: ParA (partitioning protein A), an ATPase motor protein; ParB (partitioning protein B), a centromere-binding protein, and *parS*, a centromere-like DNA site (4). The ParA can be classified into two types based on their structure and size (5): One features both a non-specific DNA (nsDNA)-binding domain and a specific DNA-binding domain, such as plasmid-encoded *Escherichia coli* P1 ParA (6); while the other one possesses a nsDNA-binding domain, such as plasmid-encoded *Streptococcus pyogenes* pSM19035 Delta (δ, *Sp*ParA), *Salmonella newport* TP228 ParF (TP228 ParA), and chromosomal-encoded *Helicobacter pylori* ParA (*Hp*ParA) (7–9). ParB comprises multiple domains, including a ParA-interacting peptide, an *N*-terminal CTPase domain involved in protein-protein interactions (NTD), a middle *parS*-binding domain (DBD), a *C*-terminal dimerization domain (CTD), and a flexible linker connecting the DBD and CTD domains (10). ParB is a dual-feature DNA-binding protein that can specifically bind to the *parS* site and non-specifically spread on the neighboring DNA (11–13). In TP228 partition system, the ParG (TP228 ParB), instead of conventional ParB, consists of a flexible *N*-terminus responsible for TP228 ParA interaction and a *C*-terminal ribbon-helix-helix domain involving in sequence-specific DNA binding (14–16).

In bacterial chromosomes, the homolog proteins of ParA and ParB are Soj (sporulation protein J) and Spo0J (stage 0 sporulation protein J), respectively. ParA forms as a dimer in the ATP-bound state that can bind to nsDNA (17,18) through a continuous basic binding patch formed by arginine/lysine residues (19,20). ParB specifically binds with *parS* forming the partition complex to mediate the paired and higher-order complex formation (21,22). The partition complex interacts with the nucleoid-bound ParA forming the nucleoprotein ParA–ParB–DNA complex, known as the nucleoid–adaptor complex (NAC) (23–26). Archaea, the third domain of life, have been characterized to be the ancestors of eukaryotes. The chromosomal segregation system, SegAB system, regulates the genome segregation of *Sulfolobus solfataricus* (27). The ParA-homolog SegA forms a novel non-sandwich dimer and exhibits two DNA binding sites. SegB specifically binds with S1 DNA and forms a higher-order partition complex. The *N*-terminal domain of SegB significantly stimulates SegA ATPase activity and architecturally regulates the segrosome (SegA–SegB–DNA) formation (28).

In bacterial ParAB*S* system, ParA is a weak ATPase and is activated by ParB and DNA in the process of DNA partitioning (7,17,18,29), such as observed in P1 ParA. P1 ParA undergoes a slow conformational change to [ParA-ATP]* upon ATP-binding, which enables its binding to non-specific DNA (30). The gradual formation of [ParA-ATP]* establishes a gradient of ParA on the nucleoid through interactions with ParB, thereby stimulating the ATPase activity of ParA. The complex comprising P1 ParA, ParB, and DNA (known as NAC) has been isolated, and the assembly and disassembly of NAC depend on the presence of ATP (25). The nsDNA binding ability of ParA, which relies on the formation of [ParA-ATP]*, is crucial for DNA partition (31,32). A key residue, aspartic acid (D41 in *Hp*ParA), coordinates the water nucleophile (W_Nu_) and initiates ATP hydrolysis, playing a significant role in ParA ATP-hydrolysis. *Tt*SojD44A, an ATP-hydrolysis deficient mutant, binds to DNA efficiently, even more so than *Tt*Soj, which dissociates from DNA time-dependently due to ATP hydrolysis (18). Further investigation is required to understand the regulatory role of ATP hydrolysis in ATP cycling and NAC formation for ParA-mediated DNA partition. ParA binds ATP to form a ParA-ATP dimer, which undergoes conformational changes to become [ParA-ATP]* dimer, enabling its binding to nsDNA (30). The [ParA-ATP]* complexes localize on the nucleoid, where it interacts with ParB-*parS* complex, forming the NAC (25). The ATP hydrolysis activity of [ParA-ATP]* is stimulated by the ParB-*parS* complex, converting [ParA-ATP]* to ParA-ADP. Upon ATP hydrolysis, ParA dissociates from the ParB–*parS* complex and releases from the nucleoid. ParA continues to cycle ATP hydrolysis, assisting in the faithful segregation of replicated DNA to the cell poles (6). ParB has been characterized as exhibiting CTPase activity which is *parS*-dependent and this function is required for regulation partition complex formation (2,33–37). Two conserved motifs, GxxRxxA and EN(I/L)QRE(D/N/E)L motifs, located in the NTD of ParB are responsible for CTP hydrolysis (34,35). Upon CTP-binding, the NTDs of the two ParB monomers undergo domain-swapped dimerization resulting in a closed conformation known as a DNA-clamp (2,33–37). The closed DNA-clamp state enables ParB dimer sliding alone the DNA and condensing the DNA efficiently (38,39).

The *Hp*ParA, *Hp*ParB and *parS* are three components of the ParAB*S* system of *Helicobacter pylori* (7). The crystal structure of the Ct-*Hp*ParB (*C*-terminal truncated) and *parS* complex reveals an elongated structure, with a flexible *N*-terminal domain for protein–protein interaction and a conserved DNA-binding domain for *parS* binding (11). The crystal structures of the *Hp*ParA in complex with ATP and DNA were previously determined, revealing its non-specific DNA binding through a lysine-rich basic binding patch and a single DNA-binding site (19). Electron microscopy studies demonstrated the potential NAC complex formation involving *Hp*ParA, *Hp*ParB and DNA in *H. pylori* chromosome partitioning system (19). Although the model organisms of chromosome segregation typically are *B. subtilis* and *C. crescentus*, the ATP-hydrolysis deficient mutant in the two species cannot be obtained for further investigations of molecular mechanism of chromosome partition (17,20). In this study, we determined the crystal structures of ATP-hydrolysis deficient mutant *Hp*ParAD41A and its DNA complex, highlighting the crucial role of Asp41 and the essential water nucleophile in the ATP hydrolysis of *Hp*ParA. Furthermore, we investigated the specific and non-specific DNA binding modes of *Hp*ParB, which are regulated by CTP. Additionally, we observed interactions among *Hp*ParA, *Hp*ParB, and DNA leading to NAC formation. Finally, we determined the ternary complex structure of *Hp*ParAD41A–DNA–*Hp*ParBN10, mimicking a potential NAC complex. The *Hp*ParAD41A–DNA–*Hp*ParBN10 complex allows us to elucidate the molecular mechanism of ParAB*S* system in chromosome partition in bacteria.

## Materials and methods

### Cloning, expression and purification of proteins

Cloning, expression and purification of *H. pylori* Soj/ParA (HP1139) and Spo0J/ParB (HP1138) have been described previously (11). Recombinant proteins were grown in Luria-Bertani medium and induced overnight at 20°C by adding 1 mM isopropyl-β-d-1-thiogalactopyranoside (IPTG). The Ni-NTA system (Cytiva) was used to purify proteins with elution buffer (20 mM Tris-HCl pH 8.0, 500 mM NaCl, 110 mM imidazole, 5 mM MgCl_2_ and 10% glycerol). The eluted proteins were applied to a Superdex™ 200 increase 10/300 size exclusion chromatography column (Cytiva) pre-equilibrated with the elution buffer and run at 0.5 ml·min^−1^. Molecular weight and purity of proteins were assessed by SDS-PAGE. *Hp*ParAD41A mutant was generated by site-directed mutagenesis method and verified by DNA sequencing and the protein purification was similar to that of *Hp*ParA (11).

### DNA substrate preparation

Double-stranded 24-bp DNA fragments were prepared using an equal molar ratio of two complementary oligonucleotides: *parS* (5’-AGGGTGTTCCACGTGAAACAGGGA-3’, with the 16-bp *parS* site underlined) and nsDNA (5’-TCCTATGAATTGCTATGGCAAGCG-3’). The DNA fragment was dissolved in a buffer containing 20 mM Tris–HCl (pH 7.5), 100 mM NaCl and 2 mM MgCl_2_. The double-stranded DNA (dsDNA) was annealed by heating to 95°C for 30 min and then gradually cooled to room temperature and stored at –20°C until use.

### *Hp*ParB *N*-terminal peptide (*Hp*ParBN10) preparation

The *N*-terminal peptide of *Hp*ParB (residue 1–10), *Hp*ParBN10, labeled with the 5-FAM-Ahx fluorophore, was synthesized and purchased from MDBio, inc. The excitation and emission wavelength of 5-FAM are 490 and 520 nm, respectively. The peptide was dissolved at a concentration of 3 mg/mL in a buffer containing 20 mM Tris–HCl (pH 8.0) and 100 mM NaCl, and it was stored at –20°C until use.

### Electrophoretic mobility shift assay

To investigate the DNA-binding mode of *Hp*ParB and the formation of NAC, electrophoretic mobility shift assay (EMSA) was conducted. For the DNA-binding mode of *Hp*ParB, reactions were performed in a 20 μl volume using a reaction buffer (20 mM Tris–HCl, pH 8.0, 50 mM NaCl, 11 mM imidazole, 5 mM MgCl_2_, 10% glycerol). *Hp*ParB or Ct-*Hp*ParB was incubated with 30 pmol *parS* or nsDNA at different molar ratios of protein to DNA (5, 10, 20 and 40), with or without 2 mM CTP. The reactions were incubated at 37°C for 15 minutes and then loaded onto a 7% acrylamide gel in Tris-glycine buffer. Electrophoresis was conducted for 1 hour and 35 minutes at 60 V and 4°C. For NAC formation, reactions were performed in a 20 μl volume in using the reaction buffer as described above. Cy3-*parS* (5 pmol) and Cy5-nsDNA (3.3 pmol) were used. *Hp*ParA and *Hp*ParB were mixed with Cy5-nsDNA and Cy3-*parS*, respectively, in a protein to DNA molar ratio of 30:1 and 20:1, with or without 2 mM CTP, and incubated at 37°C for 15 min to form [*Hp*ParA-nsDNA] and [*Hp*ParB-*parS*] complexes, separately. The pre-incubated complexes were then further incubated in a 1:1 molar ratio with or without 2 mM CTP at 37°C for 30 min. The reactions were loaded onto a 7% acrylamide gel in Tris-glycine buffer and subjected to electrophoresis for 1 h and 35 min at 60 V and 4°C and visualized by GelRed™ Nucleic Acid Gel-staining (Biotium).

### CTPase assay

CTPase activity assays of *Hp*ParB were performed by the malachite green method with some modification (40). 4 μM ParB was incubated with or without 4 μM *parS* DNA in 4 mM CTP. The reaction was in buffer 20 mM Tris–HCl (pH 8.0), 175 mM NaCl, 5 mM MgCl_2_ and 10% glycerol with the final volume of 200 μl at 37°C for 2 h. The reaction was terminated with 200 μl 10% SDS, and followed by 200 μl of 1.25% ammonium molybdate in 6.5% H_2_SO_4_, and 200 μl of 9% ascorbic acid for coloring. The hydrolyzed phosphate product and molybdic acid form a complex that can be reduced and produces a deep blue color by ascorbic acid and is monitored at 660 nm. Three independent repeats for *Hp*ParB CTPase activity were conducted with error bars representing standard deviations.

### Microscale thermophoresis

The protein-protein interaction between *Hp*ParA or *Hp*ParAD41A and *Hp*ParBN10 were measured using the microscale thermophoresis (MST) assay. Serial dilutions of unlabeled *Hp*ParA or *Hp*ParAD41A (300 μM) were prepared in a buffer (20 mM Tris–HCl (pH 8.0), 5% Glycerol, 200 mM NaCl, 50 mM Imidazole, 5 mM MgCl_2_) containing 0.05% Tween20 over 16 tubes, each containing 10 μl protein solution. Aliquots were mixed with 10 μl of 5-FAM-Ahx labeled *Hp*ParBN10 (200 nM), which was diluted to an optimal fluorescence intensity of approximately 2000 counts). Subsequently, 4 μl of each reaction mixture was loaded into premium capillaries (NanoTemper Technologies). The thermophoresis was measured at 25°C for 20 s with 40% LED power and 60% microscale thermophoresis power. The data obtained from three independent measurements were combined and analyzed using MO Affinity Analysis software (NanoTemper Technologies) to fit a binding curve.

### Fluorescence polarization binding isotherms

The equilibrium DNA binding assays with *Hp*ParA, *Hp*ParAD41A and *Hp*ParB were done by fluorescence polarization (FP) binding isotherms. The DNA substrates were fluorescently (Cy3 and Cy5) labeled on the 5’ end, which allows to measure the increase in FP of the protein-DNA complex relative to the value obtained from the protein-unbound DNA. Twenty micromolar proteins with a 2-fold serial dilution of proteins were made in 20 mM Tris–HCl (pH 8.0), 175 mM NaCl, 5 mM MgCl_2_, 10% glycerol and 1 mM ATP or CTP before being incubated with 5 nM fluorophore-labeled DNA at room temperature. DNA binding by proteins were determined by measuring the changes in fluorescence polarization using a Paradigm plate reader (Molecular Devices). The FP signal was read at 595 nm at an excitation of 535 nm and calculated by determining the concentration of protein required to bind 50% of the fluorophore-labeled DNA. The unbound state is represented by the fluorescence anisotropy of the fluorophore-labeled DNA in the presence of buffer alone. The average of three independent experiments is shown, with error bars representing standard deviations.

### Crystallization

*Hp*ParAD41A crystals were grown using *Hp*ParAD41A (5 mg/ml) with 10 mM ATP as an additive. The *Hp*ParAD41A in 20mM Tris–HCl (pH 8.0), 500 mM NaCl, 110 mM imidazole, 5 mM MgCl_2_, 10% Glycerol. The reservoir solution contained 0.1 M Tris–HCl (pH 8.5), 0.1 M MgCl_2,_ 14% PEG550MME, 16% PEG8000. The *Hp*ParAD41A crystals were obtained after 2 days incubated at 20°C and grew to a dimensions of 0.05 × 0.02 × 0.01 mm. *Hp*ParAD41A-DNA and *Hp*ParAD41A-*parS*-*Hp*ParBN10 crystals were grown using *Hp*ParA (4 mg/ml) with 10 mM ATP as an additive. The *Hp*ParAD41A in 20 mM Tris–HCl (pH 8.0), 200 mM NaCl, 44 mM Imidazole, 5 mM MgCl_2_, and 10% Glycerol were mixed with *parS* at a molar ratio of 5:1 and for the *Hp*ParAD41A-DNA-*Hp*ParBN10 crystals, *Hp*ParBN10 were added in at a molar ratio of 1:8. The mixture was incubated at 25°C for 10 min. The reservoir solution of *Hp*ParAD41-DNA and *Hp*ParAD41A-DNA-*Hp*ParBN10 contained 25% ethylene glycol and 0.1 M MES (pH 5.6), 25% ethylene glycol, respectively. The crystals were obtained after 1–2 days incubated at 25°C and grew to a dimensions of 0.3 × 0.2 × 0.2 mm.

### Data collection and structure determination

X-ray diffraction data for all the crystals used in this study were collected from beamlines TLS 15A1 and TPS 05A, National Synchrotron Radiation Research Center (NSRRC), Taiwan. All datasets were processed using HKL-2000 software (41). The structural phase was determined by molecular replacement (MR) with Phaser-MR (42), using *Hp*ParA-ATP-DNA structure (PDB ID: 6IUC) (19) as a search model. Structural refinements were performed in PHENIX (43), and structural model adjustment was carried out in COOT (44). All structural figures shown in this report were generated using PyMOL (http://pymol.org/). X-ray diffraction data and structural refinements are summarized in Table 1.

**Table 1.** X-ray diffraction data and refinement statistics

Crystal	*Hp*ParAD41A	*Hp*ParAD41A-DNA	*Hp*ParAD41A-DNA-*Hp*ParBN10
**Data collection statistics**			
Source	NSRRC-TPS05A	NSRRC-TPS05A	NSRRC-TPS05A
Wavelength (Å)	0.99984	0.99984	0.99984
Space group	P2_1_2_1_2_1_	P1	P1
Resolution (Å)	2.0	2.6	2.6
Unit cell parameter			
a (Å)	48.1	74.9	75.2
b (Å)	94.1	74.8	75.2
c (Å)	111.4	81.0	81.1
α (°)	90.0	71.3	71.3
β (°)	90.0	71.3	71.5
γ (°)	90.0	67.5	67.7
Number of reflections	1,776,973	424,572	412,260
Number of unique reflections	35,035	44,627	48,400
Redundancy of reflection	13.3	1.9	1.9
Completeness (%), overall	99.9 (100.0)^a^	96.6 (96.4)^a^	96.5 (96.2)^a^
I/σ, overall	2.4 (18.1)^a^	2.2 (16.8)^a^	2.1 (17.5)^a^
*R*_merge_^b^ (%), overall	84.8 (15.6)^a^	33.7 (4.2)^a^	32.1 (3.9)^a^
**Refinement statistics**			
Resolution (Å)	29.4-2.0	27.9-2.6	28.6-2.6
*R*-factor^c^/*R*_free_^d^ (%)	17.1/22.1	22.4/30.3	22.3/30.4
Number of reflections used	3,334	2,664	2,454
Number of residues	527	1,104	1,142
Number of atoms	4,513	9,312	9,551
Protein	4,092	8,200	8,443
DNA	-	984	984
ATP	62	124	124
Mg	2	4	4
Water	357	-	-
Average B-factor (Å^2^)	23.2	56.0	50.8
Protein	22.8	53.1	47.0
DNA	-	83.4	41.4
ATP	13.4	31.0	25.2
Mg	10.0	39.7	43.4
Water	29.4	-	-
RMSD bond lengths (Å)	0.009	0.010	0.011
RMSD bond angles (°)	1.55	1.62	1.79
PDB ID^e^	8JML	8JMK	8JMJ

^a^Values in parentheses are for the highest-resolution shell.

^b^ *R*_merge_=Σ|I−<I>|/ΣI, where I is the observed intensity and <I> is the average intensity from multiple observations of symmetry-related reflections.

^c^ *R*=Σ|F_obs_−F_calc_|/ΣF_obs_, where F_obs_ and F_calc_ are the observed and calculated structure factor amplitudes, respectively.

^d^ *R*_free_ was calculated with 5% of the total number of reflections randomly omitted from the refinement.

^e^Protein data bank identifiers for co-ordinates.

## Results

### *Hp*ParAD41A, an ATP hydrolysis-deficient mutant

**Figure 1.**
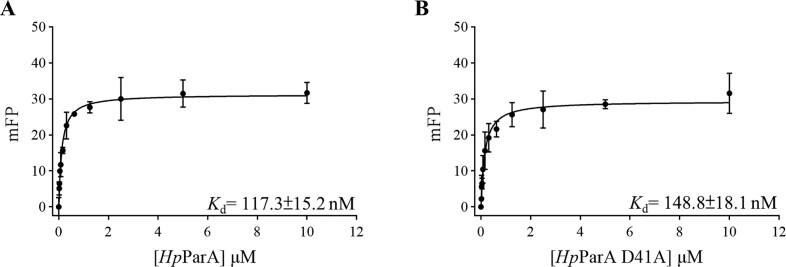
DNA Binding of *Hp*ParA and *Hp*ParAD41A. DNA binding by *Hp*ParA (**A**) and *Hp*ParAD41A (**B**) to a 24-bp nsDNA was measured by FP binding isotherms and plotted against protein concentration (0–10 μM). All measurements were reported in triplicate and error bars represent the standard deviation of the mean; the solid lines represent fitting curves to the Michaelis–Menten equation.

We have observed the formation of the nucleoid adaptor complex (NAC) by the *Hp*ParA-DNA and *Hp*ParB-*parS* complexes (19). Upon ATP hydrolysis, the *Hp*ParA-ATP dimer dissociates into *Hp*ParA monomers, resulting in the disruption of the interaction between *Hp*ParA and *Hp*ParB within the NAC. In the ParA superfamily, a conserved functional aspartic acid residue plays a critical role in ATP hydrolysis (Supplementary Figure S1A); for example, Asp44 of *Tt*Soj is known to have this function (18), and its corresponding residue in *Hp*ParA is Asp41. The side chain of *Hp*ParA Asp41 coordinates with the nucleophilic water (W_Nu_), which is crucial for initiating ATP hydrolysis and has been observed in the *Hp*ParA-ATP complex (PDB ID: 6IUB, (19)). We have utilized the ATP hydrolysis deficient mutant, *Hp*ParAD41A, to calculate the ATP hydrolysis rate, which was determined to be 0.4 ± 0.2 mol Pi released/mol *Hp*ParAD41A·hour^−1^ that was 50% lower than that of *Hp*ParA (19). We assessed the DNA-binding ability of *Hp*ParA and *Hp*ParAD41A by EMSA using a 24-bp non-specific DNA (nsDNA) (Supplementary Figure S2). We observed that both *Hp*ParA and *Hp*ParAD41A can shift the nsDNA in a concentration-dependent manner to a maximal shift at which the nsDNA was saturated with proteins. Since the mobility of the shifted bands between *Hp*ParA and *Hp*ParAD41A was different (Supplementary Figure S2), the nature of the *Hp*ParAD41A–nsDNA complex may differ from that of *Hp*ParA-nsDNA complex. We suggested that the DNA-bound competent state (ParA*_2_-ATP_2_) formation of *Hp*ParAD41A may be different from that of *Hp*ParA. Additionally, we determined the dissociation constant (*K*_d_) for DNA binding of *Hp*ParA and *Hp*ParAD41A as 117.3 ± 15.2 and 148.8 ± 18.1 nM, respectively, using fluorescence polarization (Figure 1).

### Regulation of specific and non-specific DNA binding modes of *Hp*ParB by CTP

**Figure 2.**
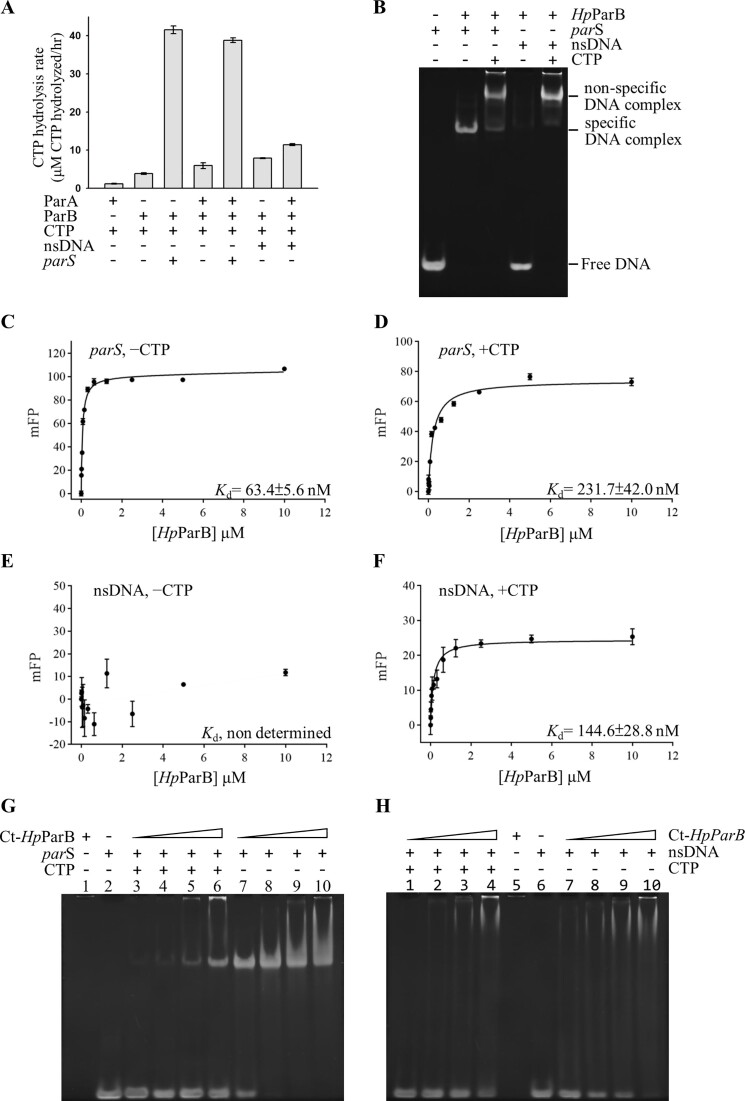
CTP hydrolysis and DNA binding of *Hp*ParB. (**A**) CTP hydrolysis of *Hp*ParB measured by colorimetric detection of inorganic phosphate using malachite green method. Error bars represent the standard error of the mean (*n* = 3). (**B**) EMSA for *Hp*ParB DNA binding. Specific and non-specific DNA binding of *Hp*ParB against *parS* and nsDNA was analyzed without or with CTP. The binding affinity of *Hp*ParB to the *parS* in the absence (**C**) and presence (**D**) of CTP was measured by FP and plotted against protein concentration (0–10 μM). The binding affinity of *Hp*ParB to the nsDNA in the absence (**E**) and presence (**F**) of CTP was measured by FP and plotted against protein concentration (0–10 μM). All measurements are reported in triplicate and error bars represent the standard deviation of the mean; the solid lines represent fitting curves to the Michaelis-Menten equation. The *parS* (**G**) and nsDNA (**H**) binding of Ct-*Hp*ParB in the absence and presence of CTP. The Ct-*Hp*ParB and DNA controls are shown in lane 1 and 2 in (**G**) and lanes 5 and 6 in (**H**), respectively.

ParB has been reported as a CTPase that binds CTP at its flexible *N*-terminal domain and catalyzes CTP hydrolysis (34,35). Two conserved motifs, GxxRxxA and ENLQRE, have been identified as participating in CTP binding and hydrolysis in *Bs*ParB (35). The corresponding CTP binding motifs, ^87^GERRLRA^93^ and ^121^ENIQRE^126^, have also been observed in *N*-terminal domain of *Hp*ParB (Supplementary Figure S1C). We assayed the CTPase activity of *Hp*ParB by the malachite green method (40). Indeed, *Hp*ParB achieved CTP hydrolysis of 3.9 ± 0.2 μM CTP/μM *Hp*ParB·hour^−1^ (Figure 2A). Moreover, the addition of *parS* notably increased CTP turnover to 10.2-folds as 41.6 ± 1.0 μM CTP/μM *Hp*ParB·hour^−1^ (Figure 2A). Upon addition of nsDNA, the CTP hydrolysis of *Hp*ParB was slightly increased to 2-folds as 7.9 ± 0.1 μM CTP/μM *Hp*ParB·hour^−1^. When adding in both the *Hp*ParA with *parS* or nsDNA, the CTPase activity of *Hp*ParB is not affected by *Hp*ParA significantly, no matter in the presence of *parS* or nsDNA. The CTPase activity of *Hp*ParB might be stimulated by *parS* specifically.

To investigate whether CTP is involved in the regulation of DNA binding by *Hp*ParB, we performed EMSA (Figure 2B) and fluorescence polarization (FP) (Figure 2C–F) using two 24-bp DNAs, *parS*-containing DNA (*parS*) and nsDNA. We observed two DNA binding modes in *Hp*ParB: specific and non-specific DNA binding. In the absence of CTP, *Hp*ParB binds *parS*, resulting in a band shift and the formation of a specific binding complex through specific DNA binding mode. However, there was only minimal binding observed with nsDNA (Figure 2B, lanes 2 and 4). The *K*_d_ for the specific DNA binding mode with *parS* in the absence of CTP was calculated as 63.4 ± 5.6 nM by FP (Figure 2C), while the binding affinity with nsDNA cannot be determined (Figure 2E). In the presence of CTP, *Hp*ParB binds with both *parS* and nsDNA, resulting in a band shift with slowly migrating mobility species, non-specific DNA complexes, which formation might be through the non-specific DNA binding mode of *Hp*ParB (Figure 2B, lane 3 and 5). The *K*_d_ values for the non-specific DNA binding mode in the presence of CTP were calculated as 231.7 ± 42.0 nM (*parS*) and 144.6 ± 28.8 nM (nsDNA) using FP (Figure 2D and F), respectively. Based on these findings, we suggest that *Hp*ParB exhibits two DNA binding modes: specific binding in the absence of CTP and non-specific binding in the presence of CTP. These results demonstrate that CTP may regulate the DNA binding mode of *Hp*ParB, when CTP-unbound *Hp*ParB showing a preference for specific DNA binding, the CTP-bound *Hp*ParB favors non-specific DNA binding. Furthermore, in the absence of CTP, the Ct-*Hp*ParB binds to *parS* forming a specific binding complex (Figure 2G, lane 7–10); however, in the presence of CTP, this complex was only observed with an excess of protein (Figure 2G, lanes 3–6). In contrast, no non-specific DNA binding complex was observed either in the presence or absence of CTP. When binding to nsDNA, neither specific nor non-specific DNA binding complexes were observed, regardless of the existence of CTP (Figure 2H, lanes 1–4 and lanes 7–10). The results demonstrated that the DBD of *Hp*ParB is responsible for specific DNA-binding; while the CTD of *Hp*ParB is involved in non-specific DNA binding.

### Interactions between *Hp*ParA, *Hp*ParB, and DNA leading to Nucleoid-adaptor complex formation

We investigated the nucleoid-adaptor complex (NAC) formation by *Hp*ParA or *Hp*ParAD41A, *Hp*ParB and DNA using EMSA (Supplementary Figure S3). The shifted bands with different mobility, NAC1 and NAC2, were observed and the amount elevated when the molar ratio of [*Hp*ParA-nsDNA]:[*Hp*ParB-*parS*] was increasing (Supplementary Figure S3A, lanes 3–5). At the same time, the amount of [*Hp*ParB-*parS*] complex vanished (Supplementary Figure S3A, lane 5). The proteins compositions of the NAC1 and NAC2 contained both *Hp*ParA and *Hp*ParB proteins that are confirmed by the peptide mass fingerprinting (PMF) (Supplementary Figure S3B). The results indicate that the [*Hp*ParB-*parS*] complex tends to interact with [*Hp*ParA-nsDNA] complex and to form NAC. The ATP hydrolysis deficient mutant, *Hp*ParAD41A, was anticipated to form a stable ATP-bound dimer than the *Hp*ParA to interact with *Hp*ParB; however, there was observed one shifted band as NAC3 when the molar ratio of [*Hp*ParAD41A-nsDNA]:[*Hp*ParB-*parS*] is increasing (Supplementary Figure S3A, lanes 7–9). Since the DNA-bound competent state (ParA*_2_-ATP_2_) formation of *Hp*ParAD41A might be slightly different as that of *Hp*ParA, the EMSA result was different from that of *Hp*ParA (Supplementary Figure S3A, lanes 5 and 9).

**Figure 3.**
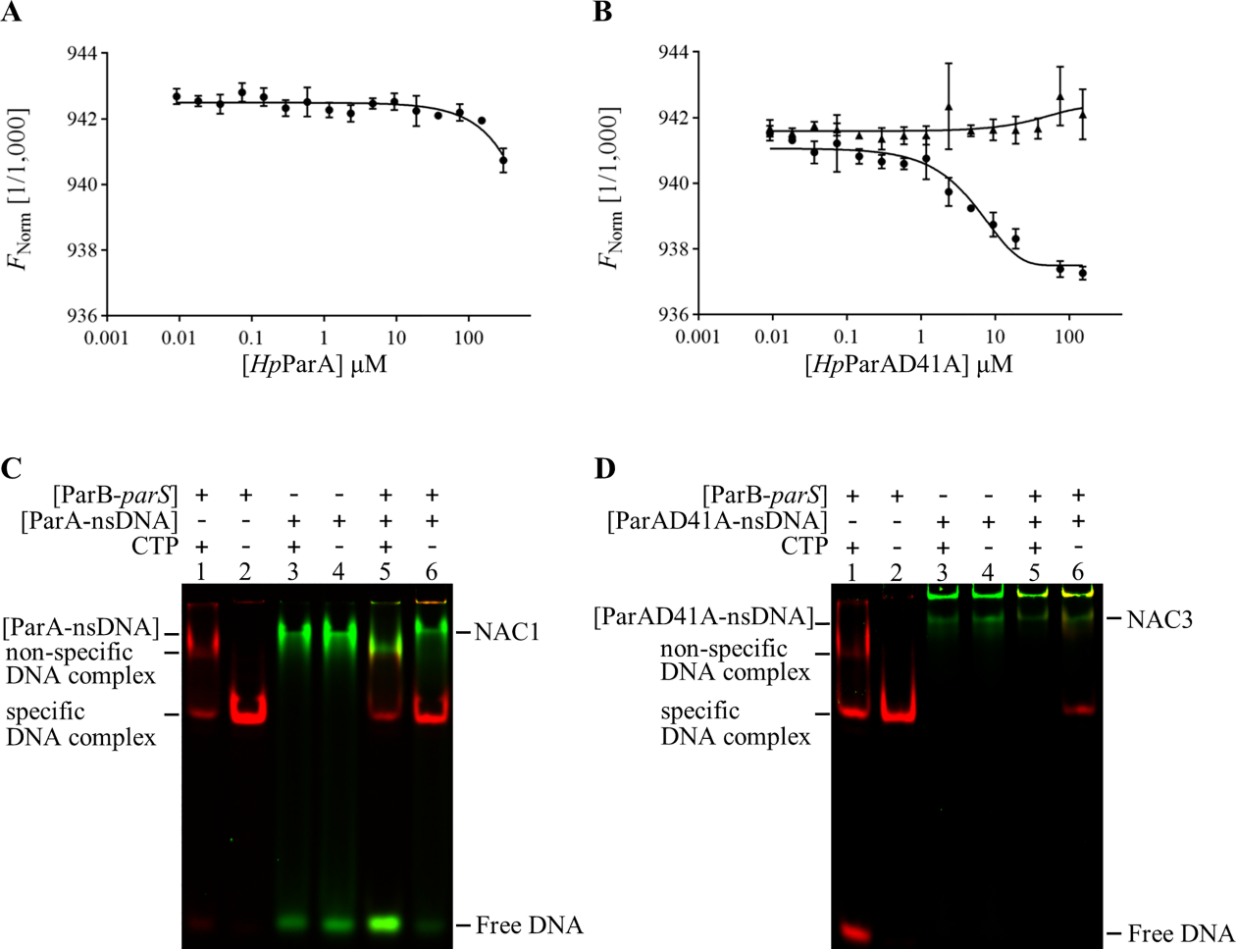
The interaction among *Hp*ParA, *Hp*ParB and DNA. Microscale thermophoresis binding measurements of *Hp*ParA and *Hp*ParAD41A with *Hp*ParB-N variants are shown in (**A**) and (**B**), respectively. (**A**) The fluorescence-labeled *Hp*ParBN10 peptide was mixed with serially diluted *Hp*ParA. (**B**) The fluorescence-labeled *Hp*ParBN10 (•) and *Hp*ParBN10R9A (▴) were mixed with serially diluted *Hp*ParAD41A. (**C**) EMSA for *Hp*ParB-*parS* complex and *Hp*ParA-nsDNA complex binding. The *Hp*ParB and *Hp*ParA were incubated with Cy3-labeled *parS* and Cy5-labeled nsDNA in the presence or absence of CTP, separately. The preincubated *Hp*ParB–*parS* complex and *Hp*ParA–nsDNA complex were further incubated together in the presence or absence of CTP and detected by EMSA. (**D**) EMSA for *Hp*ParB–*parS* complex and *Hp*ParAD41A-nsDNA complex binding. The experiments were conducted as that in (**C**) with the replacement of *Hp*ParA with *Hp*ParAD41A.

As the ParA-ATP dimer bound to DNA interacts with the *N*-terminus of ParB, we conducted protein-protein interaction measurements using Microscale Thermophoresis (MST) between the *Hp*ParA-DNA or *Hp*ParAD41A–DNA complex and the *Hp*ParBN10 peptide (residues 1–10 of *Hp*ParB) (Figure 3A and B). Both the *Hp*ParA-DNA and *Hp*ParAD41A–DNA complexes can interact with *Hp*ParBN10. However, the binding curve of *Hp*ParA does not achieve the plateau state, and the *K*_d_ cannot be determined (Figure 3A). We infer that *Hp*ParA hydrolyzes ATP and undergoes a transformation from a dimer to a monomer, resulting in the dissociation of *Hp*ParA from the DNA. The rate of this process for *Hp*ParA is faster than that of *Hp*ParAD41A. Consequently, the interaction between *Hp*ParA and *Hp*ParB is abolished, and the *K*_d_ between *Hp*ParA and *Hp*ParB cannot be determined. On the contrary, the interaction between *Hp*ParAD41A and *Hp*ParBN10 can be measured, with the calculated *K*_d_ value of 5.3 ± 1.1 μM (Figure 3B). These results revealed that the [*Hp*ParAD41A–nsDNA]:[*Hp*ParB–*parS*] complex might be energetically favorable, however, the complex formation process might be slower than that of *Hp*ParA, possibly due to the defect of DNA-bound competent state formation of *Hp*ParAD41A. The slower ATP hydrolysis rate of *Hp*ParAD41A prolongs the [*Hp*ParAD41A–nsDNA]: [*Hp*ParB-*parS*] formation and assists in the detection of NAC.

Given the potential regulation of DNA binding mode by CTP on *Hp*ParB, we conducted further investigations into the role of CTP in NAC formation using EMSA with Cy3-labeled *parS* and Cy5-labeled nsDNA (Figure 3C). We observed distinct DNA binding modes of *Hp*ParB on *parS* DNA in the absence and presence of CTP, as indicated by the presence of two shifted bands: a specific DNA complex and a non-specific DNA complex, with different mobility on the PAGE (Figure 3C, lanes 1 and 2). Specifically, *Hp*ParB exhibited specific DNA binding in the absence of CTP, while non-specific DNA binding occurred in CTP presence. In contrast, the DNA binding ability of *Hp*ParA remained unaffected by CTP (Figure 3C, lanes 3 and 4), as evidenced by the presence of shifted bands (*Hp*ParA-nsDNA) with similar mobility regardless of the existence of CTP.

Furthermore, we explored the NAC formation under the influence of CTP. In the absence of CTP, we observed that the [*Hp*ParB-*parS*] complex interact with the [*Hp*ParA-nsDNA] complex, resulting in the formation of a shifted band (NAC1) (Figure 3C, lane 6). However, in the presence of CTP, instead of NAC1, we observed a shifted band with similar mobility to that of the non-specific DNA complex of *Hp*ParB (Figure 3C, lane 5). Additionally, the amount of free DNA was greater than that of the reaction condition in the absence of CTP (Figure 3C, lane 6). For further investigations, we have performed the EMSA using the ATP-hydrolysis deficient mutant (*Hp*ParAD41A) (Figure 3D) instead of *Hp*ParA. For *Hp*ParAD41A, both with CTP and without CTP (Figure 3D, lanes 5 and 6), we observed NAC3 (nucleoid-adaptor complex 3) formation, the [*Hp*ParB-*parS*] and [*Hp*ParAD41A–nsDNA] complex. Furthermore, in the presence of CTP, the [*Hp*ParB–*parS*] complex and free DNA were not observed (Figure 3D, lane 5); however, in the absence of CTP, the [*Hp*ParB–*parS*] complex was observed but not free DNA (Figure 3D, lane 6). This result from *Hp*ParAD41A (Figure 3D) is different from that of *Hp*ParA (Figure 3C). For *Hp*ParA, in the presence of CTP, NAC1 was not observed but the [*Hp*ParB–*parS*] complex and free DNA were found. In the absence of CTP, we observed NAC1 formation, and the [*Hp*ParB–parS] complex was observed but no free DNA was left. Since the ATP hydrolysis activity of *Hp*ParAD41A is half that of *Hp*ParA, NAC3 can be observed in the presence of CTP. Because the *Hp*ParA dimer is required for DNA binding, *Hp*ParA dissociated from the nsDNA after hydrolyzing ATP; therefore, more free DNA was observed (Figure 3C, lane 5). Meanwhile, the interaction of *Hp*ParA and *Hp*ParB was abolished, and the NAC1 was not observed (Figure 3C, lane 5). We suggest that the *Hp*ParB might interact with *Hp*ParA more efficiently in the presence of CTP, promoting the ATP hydrolysis activity of *Hp*ParA. Consequently, the ATP-hydrolyzed *Hp*ParA dimer might dissociate into monomers and be released from the nsDNA, resulting in an increased amount of free DNA.

### Overall structures of *Hp*ParAD41A and the *Hp*ParAD41A-DNA complex

**Figure 4.**
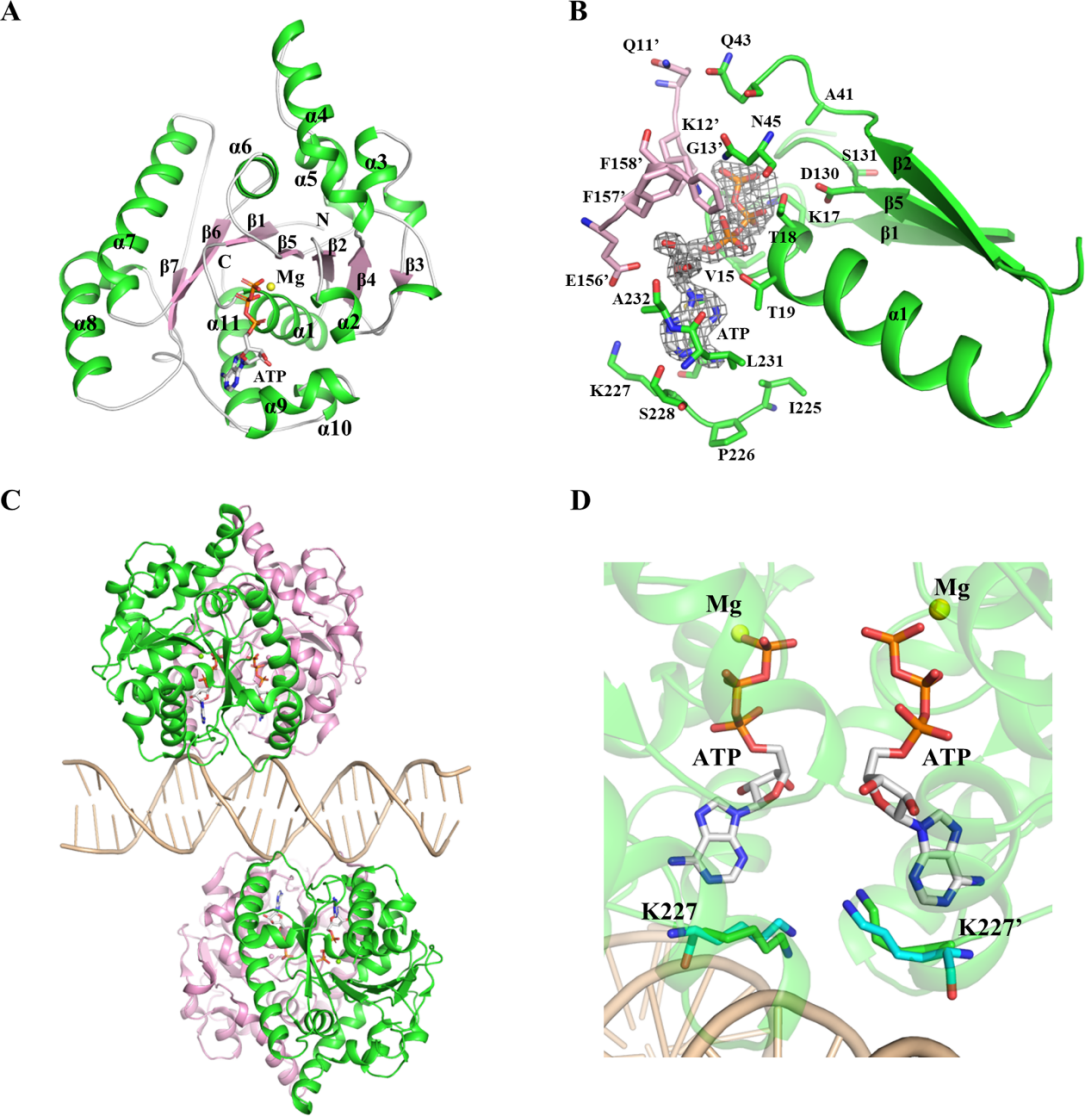
Crystal structures of *Hp*ParAD41A and its DNA complex. (**A**) *Hp*ParAD41A monomer. The structure of *Hp*ParAD41A-ATP monomer comprises eleven α-helices (α1–α11) and seven β-strands (β1–β7). ATP is shown as a stick and the magnesium ion is shown as a yellow sphere. (**B**) The ATP-binding site of the *Hp*ParAD41A-ATP complex. The *F*_o_– *F*_c_ omit electron density maps of ATP are contoured at 3.0 σ and shown as a mesh. The ATP-interaction residues from two monomers of the dimer are shown as sticks and are colored green and pink, respectively. (**C**) The *Hp*ParAD41A–DNA complex. The overall structure of the *Hp*ParAD41A-DNA complex is shown as a ribbon model. The monomers of the dimer are colored green and pink, respectively. The DNA molecule bound to the two dimers is colored wheat. (**D**) The correlated positions of K227, ATP and DNA of the *Hp*ParA-DNA and *Hp*ParAD41A–DNA complex. The DNA binding residues K227, ATP and DNA are illustrated. Residues K227 and K227’ from each monomer of the dimer are colored green and cyan for *Hp*ParA–DNA and *Hp*ParAD41A–DNA, respectively.

The water nucleophile (W_Nu_) binds to Asp41 of *Hp*ParA and interacts with the γ-phosphate of ATP to initiate ATP catalysis. To investigate the relationship between W_Nu_ and ATP hydrolysis, we solved the crystal structure of *Hp*ParAD41A in complex with ATP, *Hp*ParAD41A-ATP (Figure 4A). The overall structure of the *Hp*ParAD41A is similar to that of the *Hp*ParA (19), with a root mean square deviation (r.m.s.d.) of 0.4 Å (in Cα). In the ATP binding pocket, the electron density map of the γ-phosphate of ATP clearly revealed a complete and unhydrolyzed ATP molecule (Figure 4B). We can clearly observe the W_Nu_ located and coordinated between the γ-phosphate and Asp41 in the wild type (19), while the W_Nu_ cannot be observed in the *Hp*ParAD41A-ATP. This suggests that Asp41 likely captures the essential W_Nu_ and plays a crucial role in initiating ATP hydrolysis. As a result, *Hp*ParAD41A exhibits reduced ATP hydrolysis activity and functions as a deficient mutant in ATP hydrolysis. The detailed interactions of *Hp*ParAD41A and ATP are listed in Table 2.

**Table 2.** The interaction residues of ATP and Mg ion in *Hp*ParAD41A structure

A. ATP direct interactions							
	Monomer A			Monomer B			
Atom	Secondary structure	Residue	Atom	Secondary structure	Residue	Atom	
N1				loop α8β9	S228	N	
N6				loop α8β9	P226	O	
O3’				loop β6α7	E156	OE1	
O1A				loop β1α1	K12	NZ	
O2A	α1	T19	OG1				
	α1	T19	N				
O3A	loop β1α1	G16	N				
O1B	α1	T18	N				
	α1	T18	OG1				
O2B	loop β1α1	V15	N				
	loop β1α1	G16	N				
	α1	K17	N				
	α1	K17	NZ				
O3B	loop β1α1	G14	N				
				loop β1α1	K12	NZ	
O1G				loop β1α1	K12	NZ	
				loop β1α1	G13	N	
O2G	loop β1α1	G14	N				
	α1	K17	NZ				
**B. ATP indirect interactions**							
		**Monomer A**			**Monomer B**		
**Atom**	**Water molecules**	**Secondary structure**	**Residue**	**Atom**	**Secondary structure**	**Residue**	**Atom**
O4’	WAT473				β6	Q154	NE2
					loop β6α7	E156	OE1
					loop β6α7	E156	OE2
O3’	WAT483				loop β6α7	F157	N
		α9	A232	O			
	WAT426				loop β6α7	F158	N
O2’	WAT468	α1	T19	OG1			
		α9	L231	O			
	WAT483	α9	A232	O			
					loop β6α7	F157	N
N3	WAT614	loop α8β9	K227	NZ			
					loop β6α7	E156	OE2
O1B/O1G	WAT478		α1	D130	OD1		
			α1	D130	OD2		
			α1	S131	O		
O1G	WAT525		α1	T18	OG1		
	WAT478		α1	T18	OG1		
	WAT486		loop β2α2	Q43	OE1		
	WAT462		α2	N45	OD1		
O2G	WAT462		α2	N45	OD1		
**C. Interactions of magnesium ion**							
**Molecule**	**Atom**	**Distance (Å)**					
ATP	O1B	2.0					
	O1G	2.1					
Monomer A	Thr18 OG1	2.1					
Water	WAT437	2.1					
	WAT478	2.0					
	WAT525	2.0					

We also determined the overall structure of the *Hp*ParAD41A in complex with ATP and DNA, named *Hp*ParAD41A-DNA, that is shown in Figure 4C. In *Hp*ParA-DNA structures, four lysine residues, Lys199, Lys227, Lys230 and Lys247, have been reported to be involved in non-specific DNA binding (19), with Lys199 and Lys230 directly binding to DNA. In the *Hp*ParAD41A-DNA complex, an additional interaction with DNA involving Lys227 was observed (Figure 4D). This conserved Lys227 residue is positioned at the core of the DNA binding surface, between the DNA backbone (4.0 Å) and ATP (3.6 Å). It suggests its importance in connecting the two major functions of *Hp*ParA. Since *Hp*ParAD41A is deficient in ATP hydrolysis ability, Lys227 may interact with the DNA backbone, resulting in the loss of the connection between ATP and DNA binding.

The *Hp*ParAD41A-DNA complex displays a higher resolution of 2.6 Å compared to that of the *Hp*ParA–ATP–DNA complex structure (3.4 Å). ATP hydrolysis promotes the dissociation of the dimer into monomers. The ATP-hydrolysis deficient mutant *Hp*ParAD41A exhibits low ATP hydrolysis activity and remains in a dimeric state while preserving DNA binding. Therefore, the *Hp*ParAD41A–DNA complex may adopt a stable conformation with bound DNA than that of *Hp*ParA.

### The *Hp*ParAD41A–DNA–*Hp*ParBN10 complex, a potential nucleoid-adaptor complex

**Figure 5.**
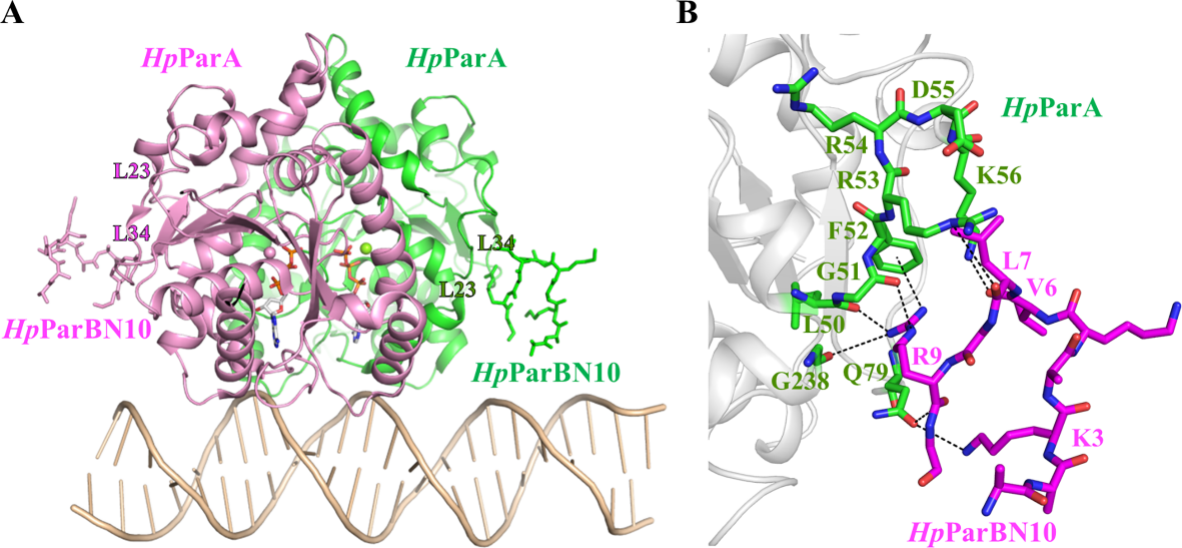
The structure of the *Hp*ParAD41A–DNA–*Hp*ParBN10 complex. (**A**) The overall structure of the *Hp*ParAD41A–DNA–*Hp*ParBN10 complex. Each monomer of the *Hp*ParAD41A dimer is shown as ribbon and colored in pink and green, respectively. The corresponding *Hp*ParBN10 peptides of each *Hp*ParAD41A monomer are shown in sticks and colored in pink and green, respectively. The DNA molecule is depicted in wheat. (**B**) The binding site of *Hp*ParBN10 in the *Hp*ParAD41A–DNA–*Hp*ParBN10 complex. The *Hp*ParBN10 is shown as sticks and colored in magenta. The *Hp*ParAD41A is shown as ribbon and colored in grey, and the residues involved in the interaction of *Hp*ParBN10 are shown as sticks, labeled and colored in green. The interactions between *Hp*ParAD41A and *Hp*ParBN10 are indicated as dashed lines.

The interaction between ParB to ParA has been mapped to the extreme *N*-terminus of ParB (45–47), as shown in Supplementary Figure S1B, which presents the *N*-terminus sequence alignment of the ParB superfamily. To investigate the molecular mechanism of NAC formation, we determined the crystal structure of the *Hp*ParAD41A–DNA in complex with residues 1–10 (^1^MAKNKVLGRG^10^) of the *Hp*ParB *N*-terminus (*Hp*ParBN10), referred to as the *Hp*ParAD41A–DNA–*Hp*ParBN10 complex (Figure 5A). The overall structure revealed that one *Hp*ParAD41A dimers binds to one 24-bps DNA, exhibiting the same architecture as the *Hp*ParAD41A–DNA complex. Additionally, each *Hp*ParAD41A binds to one *Hp*ParBN10 peptide (Figure 5A), as confirmed by the electron density map (Supplementary Figure S4). The *Hp*ParBN10 is located near the dimer interface but is inclined towards two loops, loop α2α3 (L23) and β3β4 (L34). The binding of *Hp*ParBN10 may not affect the formation of the ATP-sandwich dimer or the *Hp*ParA-DNA complex. The *Hp*ParAD41A–DNA–*Hp*ParBN10 complex suggests that the DNA-bound *Hp*ParA dimer is capable of interacting with *Hp*ParB. The *Hp*ParBN10 is clamped by the two flexible loops, L23 and L34, of *Hp*ParAD41A (Figure 5A). Although the two loops (L23 and L34) exhibit low sequence homology among ParA superfamily (residues 51–82, Supplementary Figure S1A), L23 consists mostly of charged residues (^51^GFRRDKIDYD^60^). Meanwhile, the *Hp*ParBN10 contains three positively charged residues (^1^MAKNKVLGRG^10^), leading to electrostatic interactions involved in the *Hp*ParAD41A and *Hp*ParBN10 interactions. The Lys3, Val6, Leu7 and Arg9 of *Hp*ParBN10 are the primarily interaction residues (Figure 5B), while the remaining parts are exposed to the solvent. Leu7 and Arg9 are conserved in bacterial ParB (Supplementary Figure S1B).

The *N*-terminal tail of the ParB family, which contains lysine/arginine residues, is essential for stimulating the ATPase activity of ParA (15,18,48). In *Tt*Spo0J, the Arg10 mutant fails to stimulate the ATP hydrolysis of *Tt*Soj (18). In *Bs*Spo0J, Lys3 and Lys7 play an important role in regulating the ATPase activity of *Bs*Soj (17). The corresponding residues in *Hp*ParB are Lys5 and Arg9 (Supplementary Figure S1B). Arg9 of *Hp*ParB interacts with the residues Lue50, Gly51 and Phe52 in the L23 region of *Hp*ParAD41A. Moreover, Arg9 is oriented towards the phenyl group of *Hp*ParAD41A Phe52, forming a specific cation-π interaction between the amino group and the phenyl group (Figure 5B). This interaction represents the strongest among noncovalent interactions involving a positively charged cation and negatively charged electron cloud of π systems (49). To investigate the role of the *Hp*ParB Arg9 in the interaction between *Hp*ParA and *Hp*ParB we measured the binding ability between the mutant peptide, *Hp*ParBN10R9A, and *Hp*ParAD41A using MST. The result showed that the *Hp*ParBN10R9A peptide is unable to interact with *Hp*ParAD41A as the *Hp*ParBN10 peptide (Figure 3B). The cation-π interactions between Arg9 and Phe52 may play a crucial role in the interaction between *Hp*ParB and *Hp*ParA.

Furthermore, the main chain of *Hp*ParBN10 Arg9 interacts with *Hp*ParAD41A Gln79 at L34 (Table 3), providing an additional interaction that stabilizes the *Hp*ParBN10. When superimposing the four protomers of the asymmetry unit in the *Hp*ParAD41A-DNA-*Hp*ParBN10 complex (Supplementary Figure S5A), only *Hp*ParBN10 Arg9 is located precisely in the same position, while the remaining part of *Hp*ParBN10 exhibits different conformations due to the peptide's flexibility. *Hp*ParBN10 Arg9 is firmly positioned and might play a crucial role in the interaction between *Hp*ParA and *Hp*ParB. Consequently, we propose that the key regions for the interaction between *Hp*ParA and *Hp*ParB are L23 and L34 of *Hp*ParA, and the highly conserved Arg9 of *Hp*ParB, respectively.

**Table 3.** The interactions between *Hp*ParBN10 and *Hp*ParAD41A

*Hp*ParBN10		*Hp*ParAD41A		
Residue	Atom	Residue	Atom/Group	Distance (Å)
K3	NZ	Q79	OE1	3.4
V6	O	K56	NZ	2.3
L7	O	R53	NE	3.4
R9	NH1	L50	O	2.3
	NE	G51	O	3.2
	O	Q79	OE1	3.4
	NH1	G238	O	3.7
	NH2	F52	-(C_6_H_5_)-	3.6

### Structural comparison of *Hp*ParAD41A, TP228 ParA and pNOB8 ParA complexes

**Figure 6.**
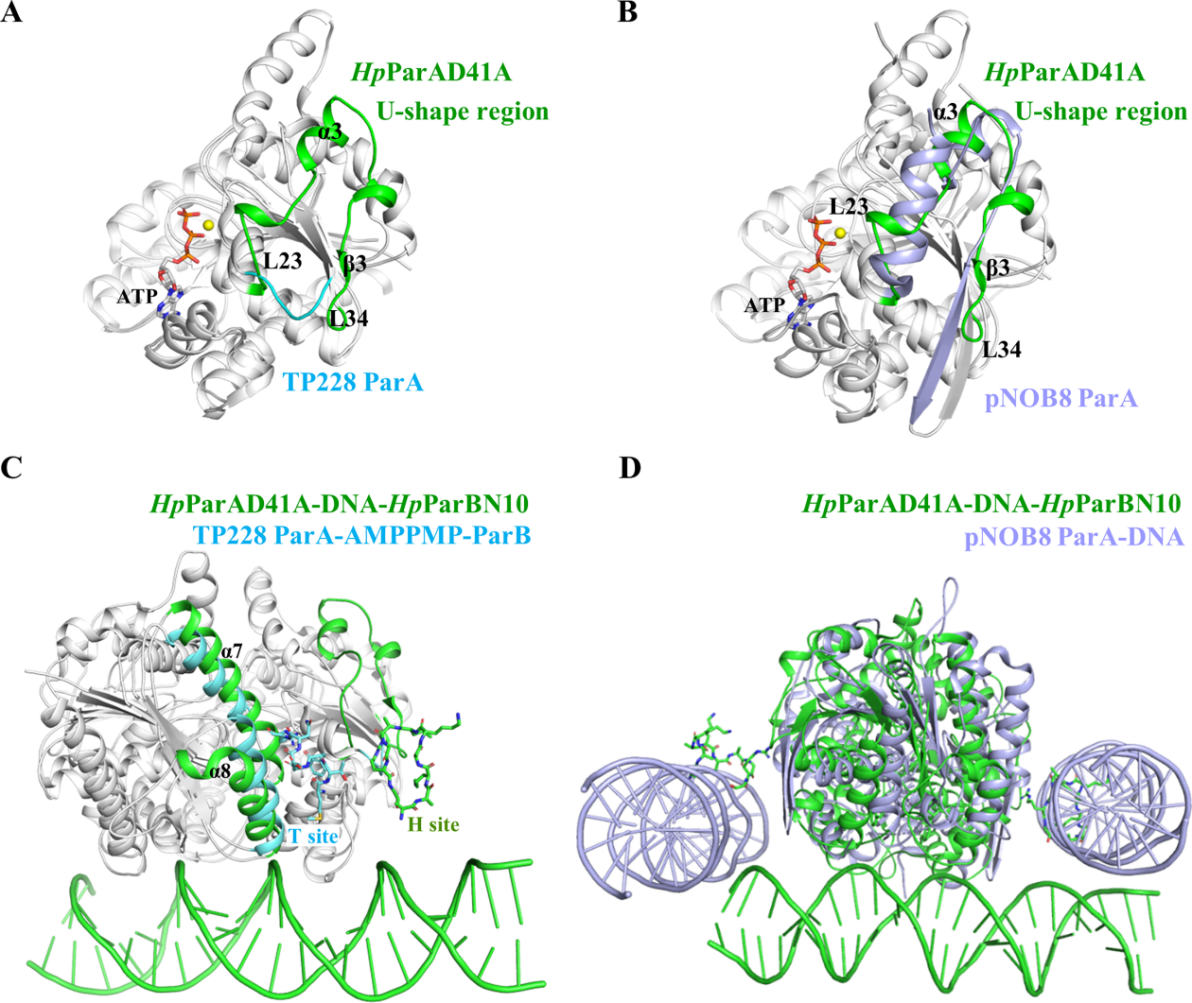
Structural comparison of *Hp*ParAD41A–DNA–*Hp*ParBN10 complex with TP228 ParA–AMPPMP–ParB and pNOB8 ParA–DNA complexes. Superimposition of the *Hp*ParAD41A monomer with TP228 ParA monomer (**A**) and pNOB8 ParA monomer (**B**), respectively. The structural differences are labeled as L23, α3, β3 and L34 in *Hp*ParAD41A (U-shape region), which are colored in green, cyan, and purple-blue for *Hp*ParAD41A, the corresponding region of TP228 ParA and pNOB8 ParA, respectively. The ATP are labeled and shown as sticks. The *Hp*ParAD41A–DNA–*Hp*ParBN10 complex is structurally superimposed with the TP228 ParA–AMPPMP–ParB complex (**C**) and pNOB8 ParA-AMPPNP-DNA complex (**D**), respectively. (**C**) The interaction region of *Hp*ParAD41A dimer and *Hp*ParBN10 is labeled as H site and colored green. The interaction region of TP228 ParA dimer and ParB fragment is labeled as T site and colored in cyan. The *Hp*ParBN10 and TP228 ParB fragments are shown as sticks and colored green and cyan, respectively. The *Hp*ParAD41A bound DNA is shown as a ribbon and colored in green. (**D**) The *Hp*ParAD41A–DNA–*Hp*ParBN10 and pNOB8 ParA–AMPPNP–DNA complexes are colored in green and purple-blue, respectively, as well as their bound DNAs are shown as ribbons and colored accordingly. The *Hp*ParBN10 are represented as sticks and colored green.

The *Hp*ParAD41A-DNA-*Hp*ParBN10 complex represents the first ParA, ParB and DNA ternary complex (NAC) within the ParAB*S* superfamily. In this study, we determined the binding sites for both *Hp*ParBN10 and DNA (Figure 5A). Another complex, the *Salmonella Newport* TP228 ParA-AMPPMP-ParB complex (PDB: 5U1G, (50)), involves ParA and ParB but lacks DNA. In the *Hp*ParAD41A and TP228 ParA complexes, the ParB fragments consists of 10 residues (*Hp*ParBN10) and 19 residues (TP228 ParBN19) peptides, respectively. The structural superimposition of the monomers from the *Hp*ParAD41A and TP228 ParA complexes is presented in Figure 6A, with an r.m.s.d. of 2.3 Å (119 of 170 Cα atoms). The *Hp*ParA and TP228 ParA monomers contain of 264 and 211 amino acids, respectively. Notably, the TP228 ParA contains a deletion in a loop region (residues 49–55), which corresponds to the loop α2α3 (L23), α3, loop α3β3, β3 and loop β3β4 (L34) (residues 51–82) in *Hp*ParAD41A, referred to as the U-shape region (Figure 6A). This U-shape region, also observed in other bacterial ParA proteins like *Tt*Soj (residues 54–80) (18) and *Sp*ParA (residues 84–116) (8), is not involved in ATP hydrolysis or DNA binding.

The structural superimposition of *Hp*ParAD41A–DNA–*Hp*ParBN10 and TP228 ParA–AMPPMP–ParB complexes shows an r.m.s.d. of 3.6 Å (281 of 367 Cα atoms) (Figure 6C). Both complexes exhibit an ATP-sandwich dimer conformation and possess a similar continuous basic patch responsible for DNA binding. In *Hp*ParA, this patch is formed by Lys199, Lys227, Lys230, Lys247, while in TP228 ParA it is formed by Asn148, Arg169, Lys174 and Lys191 (19). This suggests that *Hp*ParA and TP228 ParA likely share a similar DNA binding site (Figure 6C). The *Hp*ParBN10 binding site (named as H site) is clamped by two loops of *Hp*ParAD41A, L23 and L34 (U-shape region) (Figure 6A). In contrast, the TP228 ParB fragment binding site (named as T site) is situated near the groove of the TP228 ParA dimer interface, close to α7 and α8 (Figure 6C). Both ParB binding sites are located in the vicinity of the dimer interface but exhibit a slight displacement with a distance of 5.3 Å (Figure 6C).

In the *Hp*ParAD41A–DNA–*Hp*ParBN10 complex, Arg9 of *Hp*ParBN10 plays a direct role in the interaction between *Hp*ParA and *Hp*ParB (Figure 5A and B). The corresponding residue of Arg9 in TP228ParB is Arg19 (Supplementary Figure S1B). Arg19 has been shown to stimulate the ATPase activity of TP228 ParA and is suggested to function as an arginine finger (15). In the TP228 ParA–AMPPMP–ParB complex structure, Arg19 is positioned close to the γ-phosphate of AMPPNP, with a distance of approximately 10 Å and likely stabilizes the transition state of TP228 ParA during ATP hydrolysis (50). In the *Hp*ParAD41A–DNA–*Hp*ParBN10 complex, the distance between Arg9 of *Hp*ParBN10 and ATP is relatively far, approximately 20 Å. However, it cannot be ruled out that Arg9 serves as the arginine finger to interact with L23 (residues 50–56), which connects to the α2 (residues 45–49) (Figure 5A). Alternatively, Arg9 of *Hp*ParBN10 may simply contribute to the binding affinity between *Hp*ParA and *Hp*ParB.

The archaeal pNOB8 ParA–AMPPNP–DNA complex (PDB: 5U1J, (50)) involves ParA and DNA but lacks ParB. This complex reveals a multifaceted DNA-binding site, and each ParA dimer is surrounded by a dense DNA substrate. The structural superimposition of the *Hp*ParAD41A and pNOB8 ParA monomer is presented in Figure 6B, with an r.m.s.d. of 4.7 Å (154 of 208 Cα atoms). Additionally, the superimposition of the *Hp*ParAD41A-DNA-*Hp*ParBN10 and pNOB8 ParA–DNA structure (Figure 6D) demonstrates that the *Hp*ParA dimer binds one DNA molecule through a basic patch at the base of the dimer, while the pNOB8 ParA dimer binds two DNA molecules through a groove of basic patch at the two sides of dimer interface (Figure 6D). Therefore, the ParB binding site of *Hp*ParAD41A coincides with the DNA binding site of pNOB8 ParA. This indicates that the ParB binding site of ParA might differ between bacterial and archaeal species.

Bacterial *Hp*ParA and TP228 ParA exhibit the same DNA binding mode and possess a similar ParB interaction region near the dimer interface. However, the archaeal pNOB8 ParA adopts a different DNA binding mode, and the nature of ParB binding of archaea remains unknown, potentially differing from that in bacteria ParA. The bacterial and the archaeal ParA family likely have distinct ParB interaction regions. Base on the *Hp*ParAD41A–DNA–*Hp*ParBN10 structure, we can conclude that *Hp*ParB interacts with DNA-bound *Hp*ParA ([ParA-ATP]*). *Hp*ParB binding may stimulate the ATP hydrolysis of *Hp*ParA and trigger its dissociation from the nucleoid, thereby promoting the partitioning of the chromosome/plasmid.

## Discussion

*Hp*ParAD41A is an ATP hydrolysis deficient mutant with lower ATP hydrolysis (0.4 ± 0.2 mol Pi released/mole *Hp*ParAD41A·hour^−1^) compared to *Hp*ParA wild type (1.0 ± 0.3 moles Pi released/mole *Hp*ParA·hour^−1^) (19). We observed that *Hp*ParAD41A has a tendency to prolong the ATP-bound state when in dimer form. Additionally, we utilized fluorescence polarization to determine the DNA binding affinities of *Hp*ParA and *Hp*ParAD41A (Figure 1). The *Hp*ParAD41A mutant exhibits inferior ATP hydrolysis and DNA binding capabilities. The *Hp*ParA–DNA and *Hp*ParAD41A-DNA complexes share similar overall structures, featuring an ATP binding pocket and a readily exposed continuous basic DNA binding patch (Figure 4). Therefore, these complexes exist in a DNA-binding competent state (ParA*_2_-ATP_2_) and undergo parallel DNA binding processes. In the *Tt*Soj and the δ_2_ superfamilies, the mutants *Tt*SojD44A and the δ_2_D60A display decreased ATP hydrolysis in ATPase activity assay (8) but increased DNA binding in EMSA (18,51) compared to their wild-type counterparts. We propose that the conversion efficiency of ParA*_2_-ATP_2_ of *Hp*ParAD41A may be slower than that of *Hp*ParA, indicating that the activation energy barrier of *Hp*ParAD41A is higher than that of *Hp*ParA. Asp41 may be critical for the precise formation of the ParA*_2_-ATP_2_ conformation, necessary to overcome the energy barrier. Once the *Hp*ParAD41A-DNA complex is formed, it may maintain a more stable conformation than *Hp*ParA and undergo a steady interaction with the *N*-terminus of *Hp*ParB.

ParB can interact with both specific and non-specific DNA and both DNA binding modes are essential for chromosome partition (13,25,52,53). Our EMSA results (Figure 2B, G and H) reveal that *Hp*ParB exhibits specific DNA binding in the absence of CTP, while favoring non-specific DNA binding in the presence of CTP. The specific DNA-binding occurs via the DBD domain; whereas the non-specific DNA-binding involves in the CTD domain. In Ct-*Hp*ParB-*parS* complex, the *Hp*ParB binds specifically to DNA through residues Arg159, Asn164, Lys190, Arg215 and Glu218 located in the DBD domain (11). In the CTD domain of *Bs*ParB, a lysine-rich surface composed of K252, K255, K256 and K259 has been probed as the non-specific DNA binding surface (54). The four corresponding basic residues are K263, K267, K270 and R274 in *Hp*ParB.

The ParB superfamily undergoes conformational changes depending on the binding of CTP and *parS*, exhibiting open and closed conformations (33,36). In the Ct-*Hp*ParB–*parS* complex without CTP, α3 in the NTD and α4 in the DBD fold as a hairpin and the NTDs of ParB dimer do not occur domain-swapping, resulting in an open conformation. Conversely, in the *Bs*ParB–CDP complex, α3 swings out by 103.1º (Supplementary Figure S5B) and the NTDs of ParB dimer undergo domain-swapping, resulting in a closed conformation. Previous reports have indicated that in the native state of ParB, the predominant orientation of the NTD and DBD is tethered together. Additionally, we observed that the addition of *parS* enhances the CTPase activity of *Hp*ParB (Figure 2A), similar to that is observed in *Bs*ParB. We propose that *parS* binding of ParB serves two functional roles in the ParAB*S* system: (i) loosening the NTD to facilitate domain-swapping and CTP binding, thus promoting the formation of the closed conformation and (ii) assisting in exposing the extreme *N*-terminus of ParB, allowing it to interact with the ParA–DNA complex.

The ParB superfamily comprises three domains: NTD, DBD, and CTD domains, each with unique functions, including ParA interaction/CTP-binding, *parS* binding, and dimerization/non-specific DNA binding, respectively. The NTD and DBD domains are connected by α3, whose orientation could be regulated by CTP binding, determining the open or closed conformation of ParB. A flexible linker (residues 233–245 in *Hp*ParB), with varying consensus among the ParB superfamily, aids in building a DNA-storing chamber for non-specific DNA binding between the DBD and CTD domains. The CTP molecule acts as a molecular switch, controlling the DNA binding mode during chromosome partition.

Furthermore, we determined the crystal structure of the *Hp*ParAD41A–DNA–*Hp*ParBN10 complex, which mimics the nucleoid-associated complex (NAC), and the *Hp*ParBN10 peptide is positioned near the dimer interface of *Hp*ParAD41A without interfering with its ATP and DNA binding site. However, the ATP-bound and DNA-bound ParA dimer is required for ParB interaction. Previously, in the TP228 ParA–AMPPNP–ParB complex (PDB: 5U1G) (50), ParA is in the AMPPNP-bound state without DNA, interacting with the ParB *N*-terminus fragment (residues 15–23) and it has suggested that Arg19 of TP228 ParB functions as an arginine finger, stimulating the ATPase activity of ParA (15). Both ParB binding sites are located near the dimer interface, however, *Hp*ParA at H site in the U-shape region and TP228 ParA at T site around α7–α8. In the *Hp*ParAD41A–DNA–*Hp*ParBN10 complex, the corresponding residue of Arg19 is conserved Arg9 in *Hp*ParBN10, which interacts with *Hp*ParAD41A Phe52 through a novel cation–π interaction which may be significant for *Hp*ParA and *Hp*ParB interaction. Bacterial *Hp*ParA and TP228 ParA exhibit the same DNA binding mode and possess a similar ParB interaction region near the dimer interface. However, the archaeal pNOB8 ParA adopts a different DNA binding mode, the ParB binding site of *Hp*ParAD41A coincides with the DNA binding site of pNOB8 ParA. This indicates that the ParB binding site of ParA might differ between bacterial and archaeal.

**Figure 7.**
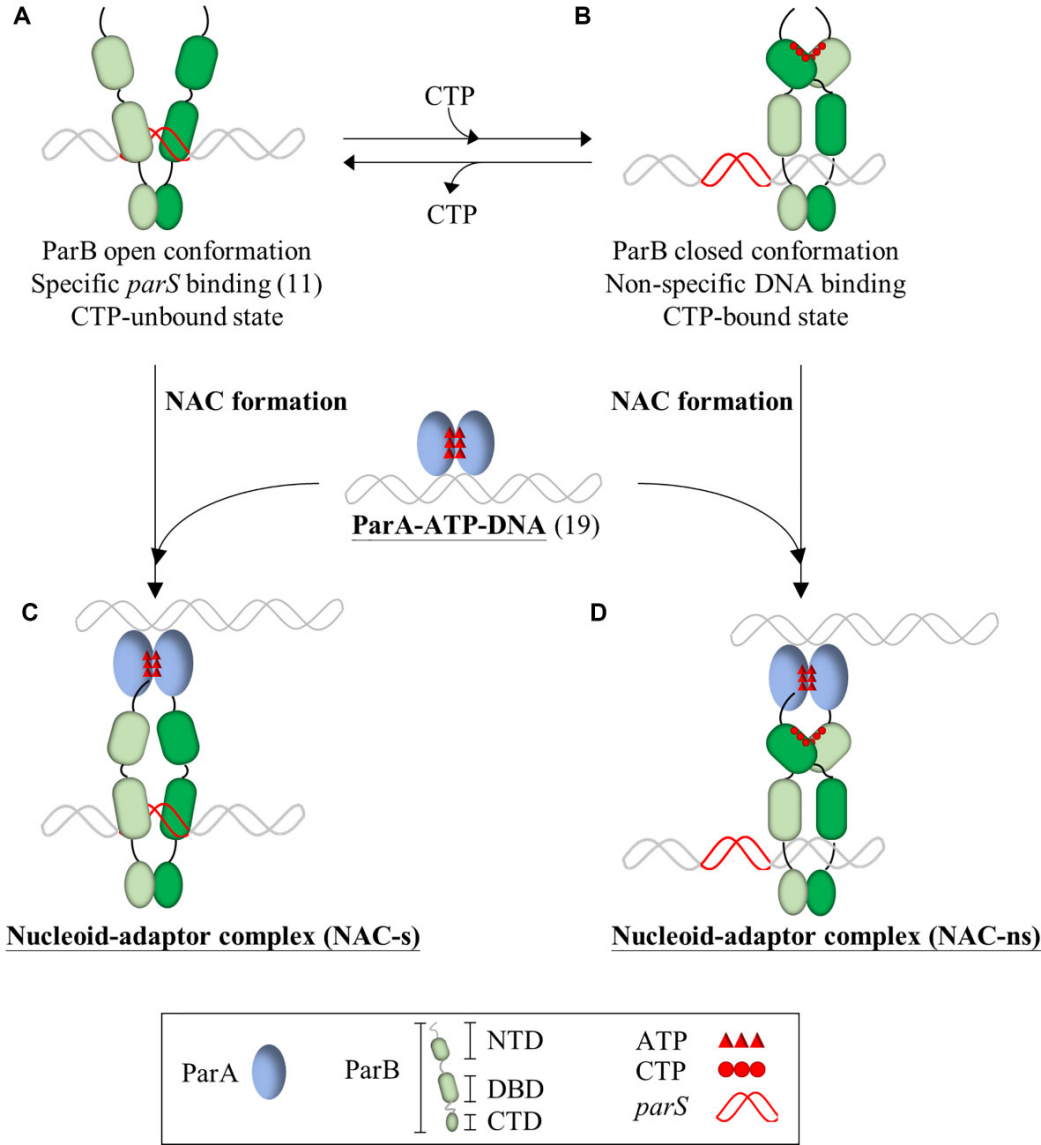
Molecular mechanism model of ParAB*S* system in chromosome partition. The ParA and ParB proteins in the ParAB*S* system exhibit distinct functional states and interactions. ParB is composed of three domains: the *N*-terminal domain (NTD), the DNA binding domain (DBD), and the *C*-terminal domain (CTD) and colored green. Specific DNA binding mode in open conformation and non-specific DNA binding mode in closed conformation of ParB regulated by CTP are shown in (**A**) and (**B**), respectively. The ParA-ATP-DNA complex is shown and colored purple-blue. Both the specific DNA complex ParB-*parS* (**A**) and non-specific DNA complex ParB-DNA (**B**) might be interacted with ParA-ATP-DNA to form the nucleoid-adaptor complex (NAC), NAC-s (**C**) and NAC-ns (**D**), respectively.

Based on these results, we propose a molecular mechanism model of ParA, ParB and *parS* in the ParAB*S* system during chromosome partition (Figure 7). In the CTP-unbound state (Figure 7A), ParB adopts an open conformation and specifically binds with *parS*. One of the two conserved CTP-binding motifs, GxxRxxA, plays an important role in molecular interactions, enabling ParB to spread, bridge, and condense DNA after binding to *parS*. In the CTP-bound state (Figure 7B), ParB assumes a closed conformation and binds non-specifically to DNA and allows ParB to entrap and slide along the distal region from the *parS* site on the chromosomal DNA. ParB binds to *parS* not only facilitates CTP binding and promotes the formation of the closed conformation but also assists in exposing the extreme *N*-terminus of ParB, allowing it to interact with the ParA–ATP–DNA complex. Upon exposure of the *N*-terminus, both the *parS*-bound (Figure 7A) and nsDNA-bound (Figure 7B) of ParBs can interact with ParA through the novel cation-π interaction between a conserved Arg residue of the *N*-terminal ParB and a hydrophobic residue of ParA (for example Phe52 in *Hp*ParA). The ParA–ATP–DNA complex competently interacts with *parS*-bound or nsDNA-bound ParB to form the NAC complexes, NAC-s (Figure 7C) and NAC-ns, (Figure 7D), respectively. In the presence of CTP, the non-specific DNA-bound ParB might interact more proficiently with the ParA-ATP-DNA than in the absence of CTP, promoting ATP hydrolysis and causing ParA to dissociate from a dimer to a monomer, thus releasing the nucleoid DNA (Figure 7D). In summary, CTP regulates the DNA binding modes of *Hp*ParB and the interaction of *Hp*ParA and *Hp*ParB, and the crystal structure of the *Hp*ParAD41A–DNA–*Hp*ParBN10 complex mimics the NAC formation, providing insight into the molecular mechanism of the ParAB*S* system in bacterial chromosome partition.

## Supplementary Material

gkae450_Supplemental_File

## Data Availability

The atomic coordinates and structure factors have been deposited in the Protein Data Bank (http://www.rcsb.org/pdb) with PDB ID codes 8JML (*Hp*SojD41A-ATP), 8JMK (*Hp*SojD41A-ATP-DNA) and 8JMJ (*Hp*SojD41A-DNA-*Hp*Spo0JN10).
