# Supplementary material for: Insights into the molecular mechanism of ParABS system in chromosome partition by HpParA and HpParB
Source: Nucleic Acids Res. 2024 Jun 6;52(12):7321–36. doi: 10.1093/nar/gkae450 (PMC11229316; doi:10.1093/nar/gkae450)
Supplement: gkae450_Supplemental_File [file gkae450_supplemental_file.pdf]

A

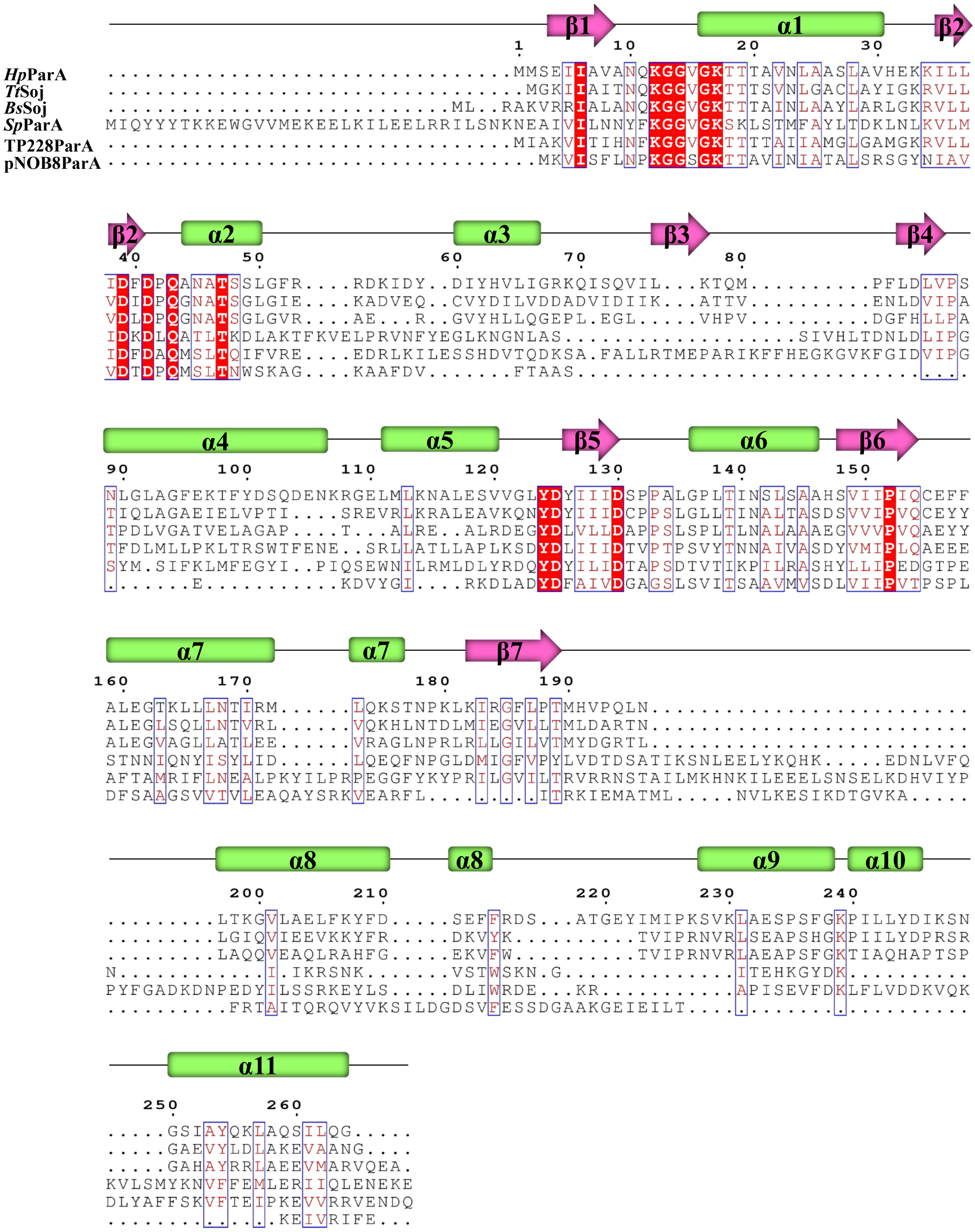
β1
α1
β2
1
10
20
30
HpParA
TtSoj
BsSoj
SpParA
TP228ParA
pNOB8ParA
α2
α3
β3
β4
40
50
60
70
80
α4
α5
β5
α6
β6
90
100
110
120
130
140
150
α7
α7
β7
160
170
180
190
α8
α8
α9
α10
200
210
220
230
240
α11
250
260

B

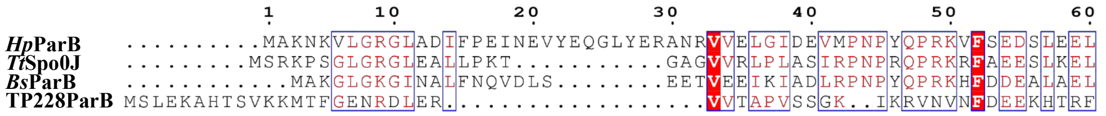
1 10 20 30 40 50 60
HpParB ...........MAKNKVLGRGLADIFPEINEVYEQGLYERANRVVELGIDEVMPNPYQPRKVFSEDSLEEL
TtSpo0J ..........MSRKPSGLGRGLEALLPKT............GAGVVRLPLASIRPNPRQPRKRFAEESLKEL
BsParB .............MAKGLGKGINALFNQVDLS........EETVEEIKIADLRPNPYQPRKHFDDEALAEL
TP228ParB MSLEKAHTSVKKMTFGENRDLER...................VVTAPVSSGK..IKRVNVNFDEEKHTRF

C

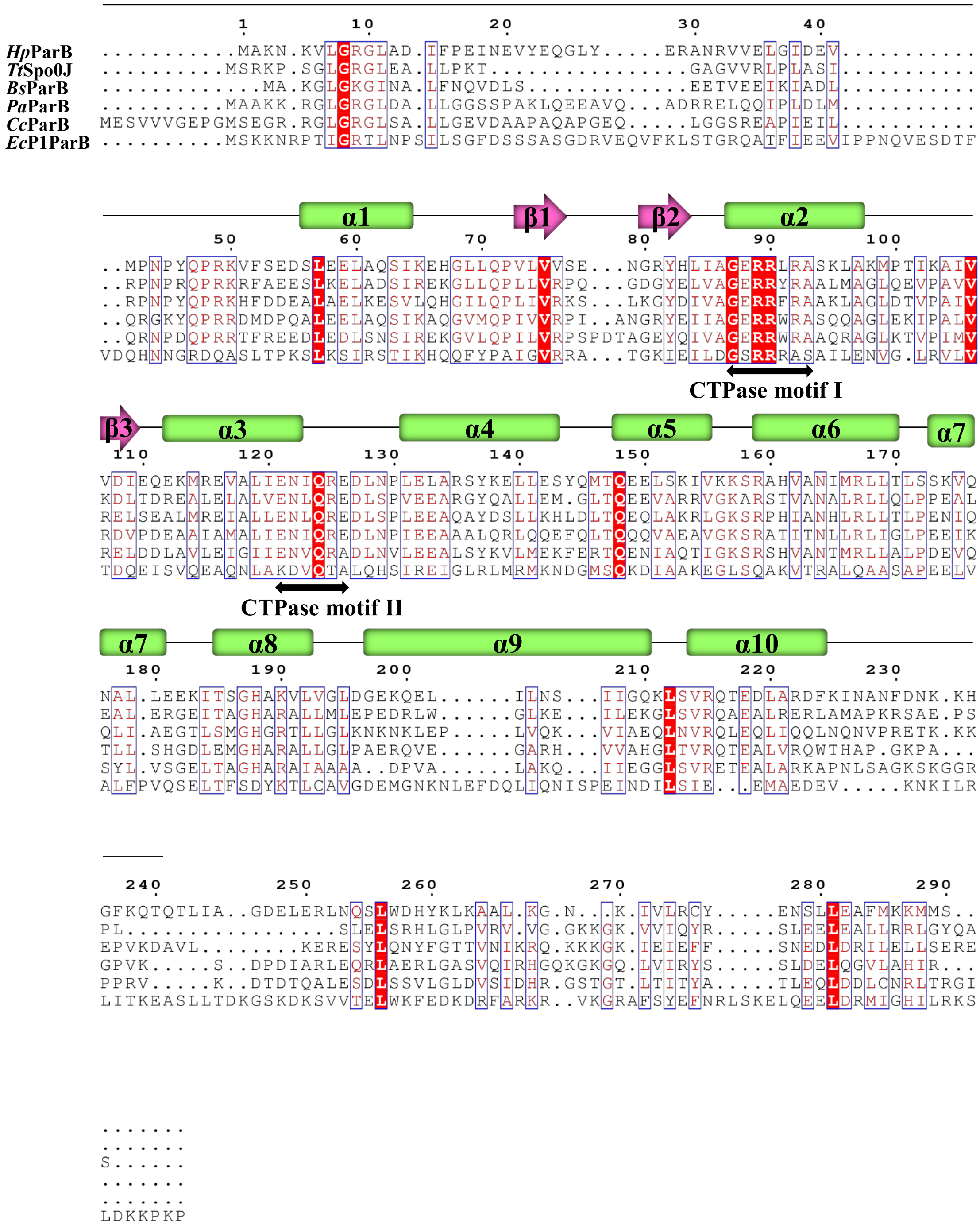
1 10 20 30 40
HpParB .............MAKN.KVLGRGLAD.IFPEINEVYEQGLY.....ERANRVVELGIDEV...........
TtSpo0J ..........MSRKP.SGLGRGLEA.LLPKT..................GAGVVRLPLASI...........
BsParB .............MA.KGLGKGINA.LFNQVDLS..............EETVEEIKIADL...........
PaParB ..........MAAKK.RGLGRGLDA.LLGGSSPAKLQEEAVQ...ADRRELQQIPLDLM...........
CcParB MESVVVGEPGMSEGR.RGLGRGLSA.LLGEVDAAPAQAPGEQ.....LGGSREAPIEIL...........
EcP1ParB ..........MSKKNRPTIGRTLNPSILSGFDSSSASGDRVEQVFKLSTGRQATFIEEVIPPNQVESDTF
α1
β1
β2
α2
50 60 70 80 90 100
..MPNPYQPRKVFSEDSLEELAQSIKEHGLLQPVLVVSE...NGRYHLIAGERRLRASKLAKMPTIKAIV
..RPNPRQPRKRFAEESLKELADSIREKGLLQPLLVRPQ...GDGYELVAGERRYRAALMAGLQEVPAVV
..RPNPYQPRKHFDDEALAELKESVLQHGILQPLIVRKS...LKGYDIVAGERRFRAAKLAGLDTVPAIV
..QRGKYQPRRDMDPQALEELAQSIKAQGVMQPIVVRPI..ANGRYEIIAGERRWRASQQAGLEKIPALV
..QRNPDQPRRTFREEDLEDLSNSIREKGVLQPILVRPSPDTAGEYQIVAGERRWRAAQRAGLKTVPIMV
VDQHNNGRDQASLTPKSLKSIRSTIKHQQFYPAIGVRRA...TGKIEILDGSRRRASAILENVG.LRVLV
CTPase motif I
β3
α3
α4
α5
α6
α7
110 120 130 140 150 160 170
VDIEQEKMREVALIENIQREDLNPLELARSYKELLESYQMTQEELSKIVKKSRAHVANIMRLLTLSSKVQ
KDLTDREALELALVENLQREDLSPVEEARGYQALLEM.GLTQEEVARRVGKARSTVANALRLLQLPPEAL
RELSEALMREIALLENLQREDLSPLEEAQAYDSLLKHLDLTQEQLAKRLGKSRPHIANHLRLLTLPENIQ
RDVPDEAAIAMALIENIQREDLNPIEEAAALQRLQQEFQLTQQQVAEAVGKSRATITNLLRLIGLPEEIK
RELDDLAVLEIGIIENVQRADLNVLEEALSYKVLMEKFERTQENIAQTIGKSRSHVANTMRLLALPDEVQ
TDQEISVQEAQNLAKDVQTALQHSIREIGLRLMRMKNDGMSQKDIAAKEGLSQAKVTRALQAASAPEELV
CTPase motif II
α7
α8
α9
α10
180 190 200 210 220 230
NAL.LEEKITSGHAKVLVGLDGEKQEL......ILNS...IIGQKLSVRQTEDLARDFKINANFDNK.KH
EAL.ERGEITAGHARALLMLEPEDRLW......GLKE...ILEKGLSVRQAEALRERLAMAPKRSAE.PS
QLI.AEGTLSMGHGRTLLGLKNKNKLEP.....LVQK...VIAEQLNVRQLEQLIQQLNQNVPRETK.KK
TLL.SHGDLEMGHARALLGLPAERQVE......GARH...VVAHGLTVRQTEALVRQWTHAP.GKPA...
SYL.VSGELTAGHARAIAAAA..DPVA......LAKQ...IIEGGLSVRETEALARKAPNLSAGKSKGGR
ALFPVQSELTFSDYKTLCAVGDEMGNKNLEFDQLIQNISPEINDILSIE...EMAEDEV.....KNKILR
240 250 260 270 280 290
GFKQTQTLIA..GDELERLNQSLWDHYKLKAAL.KG.N...K.IVLRCY.....ENSLLEAFMKKMMS..
PL.................SLELSRHLGLPVRV.VG.GKKGK.VVIQYR.....SLEELEALLRRLGYQA
EPVKDAVL........KERESYLQNYFGTTVNIKRQ.KKKGK.IEIEFF.....SNEDLDRILELLSERE
GPVK.....S..DPDIARLEQRLAERLGASVQIRHGQKGKGQ.LVIRYS.....SLDELQGVLAHIR...
PPRV.....K..DTDTQALESDLSSVLGLDVSIDHR.GSTGT.LTITYA.....TLEQLDDLCNRLTRGI
LITKEASLLTDKGSKDKSVVTELWKFEDKDRFARKR..VKGRAFSYEFNRLSKELQEELDRMIGHILRKS
....
....
S......
....
....
LDKKPKP

Supplementary Figure S1. Multiple sequence alignment of ParA/Soj and ParB/Spo0J superfamily. (A) Multiple sequence alignments of the ParA/Soj superfamily members from *Helicobacter pylori* soj (*Hp*ParA), *Thermus thermophilus* soj (*Tt*Soj), *Bacillus subtilis* soj (*Bs*Soj), *Streptococcus pyogenes* pSM19035 Delta (*Sp*ParA), *Samonella newport* TP228 ParA (TP228 ParA) and *Sulfolobus* NOB8H2 pNOB8 ParA (pNOB8 ParA) are shown. Fully conserved residues are shaded in red, and regions with similar residues are boxed in blue. Secondary-structure elements of *Hp*Soj are as cylinders and arrows for its α-helices (α1-11) and β-strands (β1-7), respectively. The residue numbering uses that of *Hp*Soj. (B) Multiple sequence alignments of the N-terminus of ParB/Spo0J superfamily members from *Helicobacter pylori* Spo0J (*Hp*ParB), *Thermus thermophilus* Spo0J (*Tt*Spo0J), *Bacillus subtilis* Spo0J (*Bs*ParB), and *Samonella newport* TP228 ParA (TP228 ParB) are shown. (C) Multiple sequence alignments of the ParB/Spo0J superfamily members from *Hp*Spo0J, *Tt*Spo0J, *Bs*ParB, *Pseudomonas aeruginosa* ParB (*Pa*ParB), *Caulobacter crescentus* ParB (*Cc*ParB) and *Escherichia coli* P1 ParB (P1 ParB) are shown. Fully conserved residues are shaded in red, and regions with similar residues are boxed in blue. Secondary-structure elements of *Hp*Spo0J are as cylinders and arrows for its α-helices (α1-10) and β-strands (β1-3), respectively. CTPase motif I (G87-A93) and II (E121-E126) are indicated.

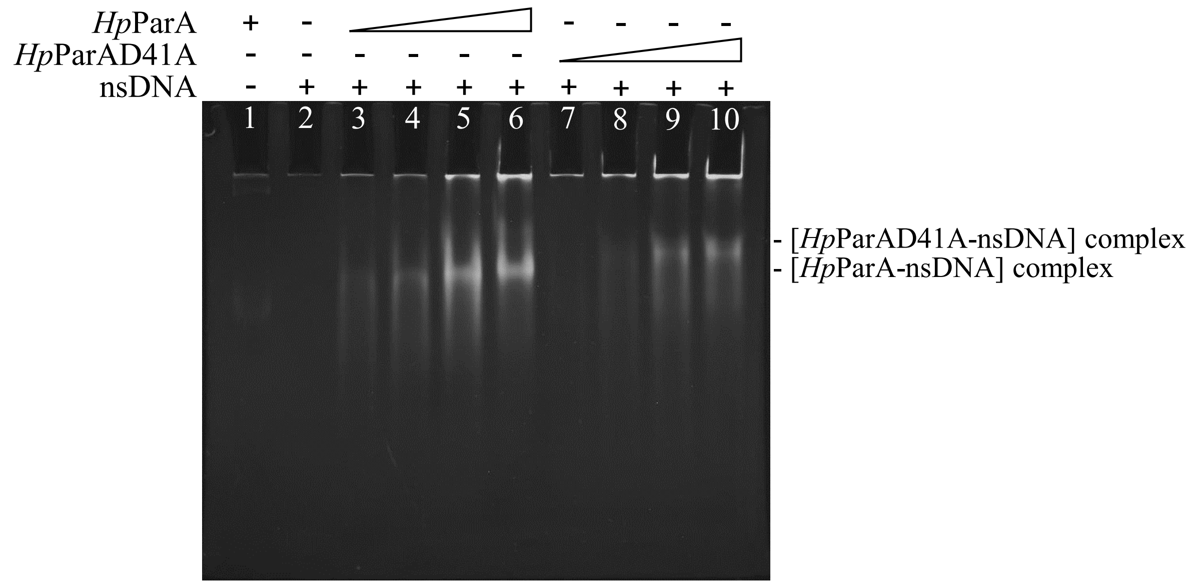

Supplementary Figure S2. EMSA of the nsDNA binding of *Hp*ParA and *Hp*ParAD41A. The *Hp*ParA (lane 3 to 6) and *Hp*ParAD41A (lane 7 to 10) bind nsDNA in a concentration-dependent fashion. Lane 1 and 2 are *Hp*ParA and free nsDNA as controls, respectively. For observation of the band shift difference between *Hp*ParA-nsDNA and *Hp*ParAD41A-nsDNA complexes, the electrophoresis time for free nsDNA has moved outside the PAGE.

A

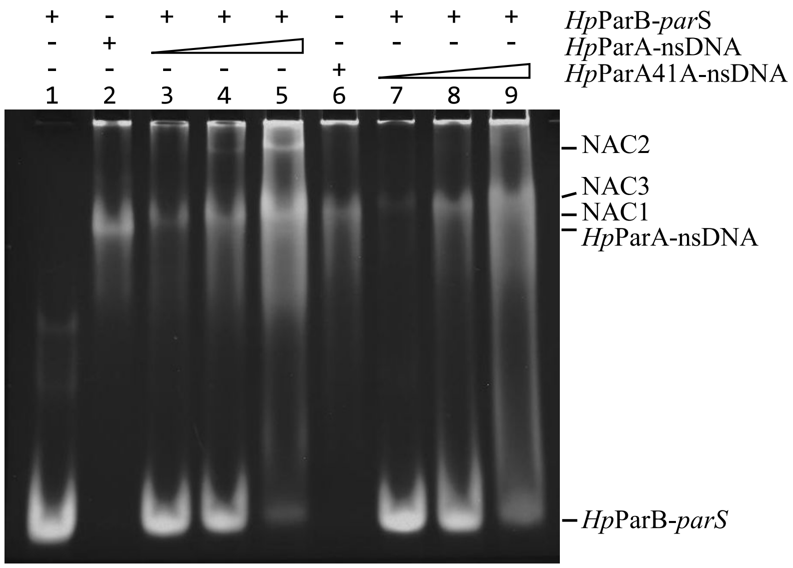

B

PMF index of NAC_1

	Accession	Mass	Score	Description
1.	Mixture 1		223	WP_000986738.1 + WP_001107428.1
2.	WP 000986738.1	29325	178	WP_000986738.1 chromosome partitioning ATPase Soj [Helicobacter pylori]
3.	WP 001107428.1	33414	86	WP_001107428.1 ParB/RepB/Spo0J family partition protein [Helicobacter pylori]
4.	WP 013356191.1	13652	31	WP_013356191.1 hypothetical protein [Helicobacter pylori]
5.	WP 001263329.1	32890	27	WP_001263329.1 glycosyltransferase family 25 protein [Helicobacter pylori]
6.	WP 000251162.1	35717	27	WP_000251162.1 chemotaxis protein [Helicobacter pylori]
7.	WP 000469341.1	29368	24	WP_000469341.1 TatD family hydrolase [Helicobacter pylori]
8.	WP 000813729.1	15313	24	WP_000813729.1 nucleoside-diphosphate kinase [Helicobacter pylori]
9.	WP 000778325.1	85629	23	WP_000778325.1 phenylalanine--tRNA ligase subunit beta [Helicobacter pylori]
10.	WP 001212434.1	52243	23	WP_001212434.1 acid-sensing histidine kinase ArsS [Helicobacter pylori]

PMF index of NAC_2

	Accession	Mass	Score	Description
1.	WP 000986738.1	29325	180	WP_000986738.1 chromosome partitioning ATPase Soj [Helicobacter pylori]
2.	WP 001107428.1	33414	67	WP_001107428.1 ParB/RepB/Spo0J family partition protein [Helicobacter pylori]
3.	WP 001213216.1	25954	26	WP_001213216.1 16S rRNA (uracil(1498)-N(3))-methyltransferase [Helicobacter pylori]
4.	WP 000991174.1	12657	23	WP_000991174.1 30S ribosome-binding factor RbfA [Helicobacter pylori]
5.	WP 000251162.1	35717	22	WP_000251162.1 chemotaxis protein [Helicobacter pylori]
6.	WP 000889791.1	58355	20	WP_000889791.1 ABC transporter ATP-binding protein [Helicobacter pylori]
7.	WP 014537154.1	7460	19	WP_014537154.1 flagellar biosynthesis anti-sigma factor FlgM [Helicobacter pylori]
8.	WP 014537104.1	19427	19	WP_014537104.1 methylated-DNA--[protein]-cysteine S-methyltransferase [Helicobacter pylori]
9.	WP 001161913.1	45844	19	WP_001161913.1 type IV secretion system protein [Helicobacter pylori]
10.	WP 000924932.1	42655	18	WP_000924932.1 dihydroorotase family protein [Helicobacter pylori]

PMF index of NAC_3

	Accession	Mass	Score	Description
1.	Mixture 1		175	WP_000986738.1 + WP_001107428.1
2.	WP 000986738.1	29325	161	WP_000986738.1 chromosome partitioning ATPase Soj [Helicobacter pylori]
3.	WP 001107428.1	33414	68	WP_001107428.1 ParB/RepB/Spo0J family partition protein [Helicobacter pylori]
4.	WP 001170531.1	50756	39	WP_001170531.1 hypothetical protein [Helicobacter pylori]
5.	WP 000924932.1	42655	33	WP_000924932.1 dihydroorotase family protein [Helicobacter pylori]
6.	WP 001213216.1	25954	32	WP_001213216.1 16S rRNA (uracil(1498)-N(3))-methyltransferase [Helicobacter pylori]
7.	WP 000546936.1	23482	28	WP_000546936.1 GIY-YIG nuclease family protein [Helicobacter pylori]
8.	WP 001155704.1	20429	23	WP_001155704.1 hypothetical protein [Helicobacter pylori]
9.	WP 014537164.1	40050	21	WP_014537164.1 radical SAM family heme chaperone HemW [Helicobacter pylori]
10.	WP 000778325.1	85629	20	WP_000778325.1 phenylalanine--tRNA ligase subunit beta [Helicobacter pylori]

C

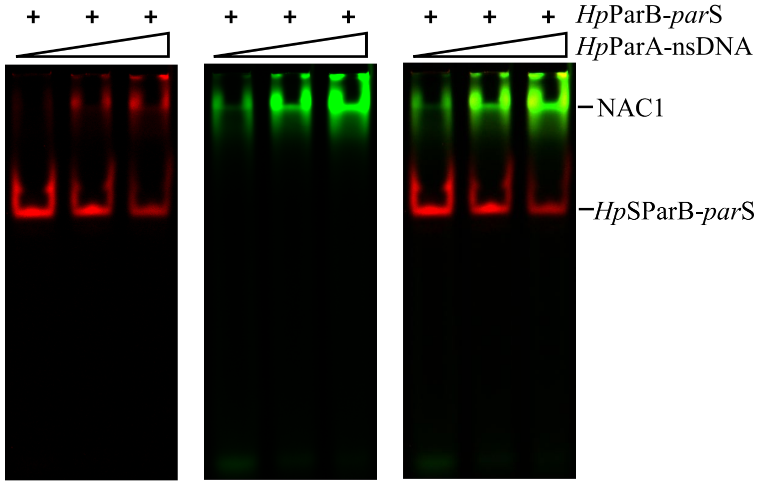

Supplementary Figure S3. The NAC complexes formed by *Hp*ParA/*Hp*ParAD41A-nsDNA and *Hp*ParB-*parS* complexes analyzed by EMSA. (A) The pre-formed *Hp*ParB-*parS* complexes (lane 1) were incubated with increasing molar ratio (1:1, 1:2, and 1:4) of the pre-formed *Hp*ParA-nsDNA (lane 3, 4, and 5) and *Hp*ParAD41A-nsDNA (lane 7, 8, and 9) complexes, respectively. The pre-formed *Hp*ParA-nsDNA and *Hp*ParAD41A-nsDNA complexes are shown in lanes 2 and 6 as controls. The putative NAC formed by *Hp*ParB-*parS* with *Hp*ParA-nsDNA and *Hp*ParAD41A-nsDNA complexes are labeled as NAC1, NAC2, and NAC3, respectively. (B) Peptide mass fingerprinting (PMF) results. The protein compositions of the band shifts from the putative NAC 1, NAC 2, and NAC 3 were analyzed by protein fingerprinting. The numerous masses observed were sufficient to identify the proteins as *Hp*ParA and *Hp*ParB from each band (red boxes). (C) The NAC1 formed by *Hp*ParB-*par*S (Cy3-labeled) and *Hp*ParA-nsDNA (Cy5-labeled) analyzed by EMSA. The NAC1 band showed both Cy3- and Cy5- fluorescence signals.

A B

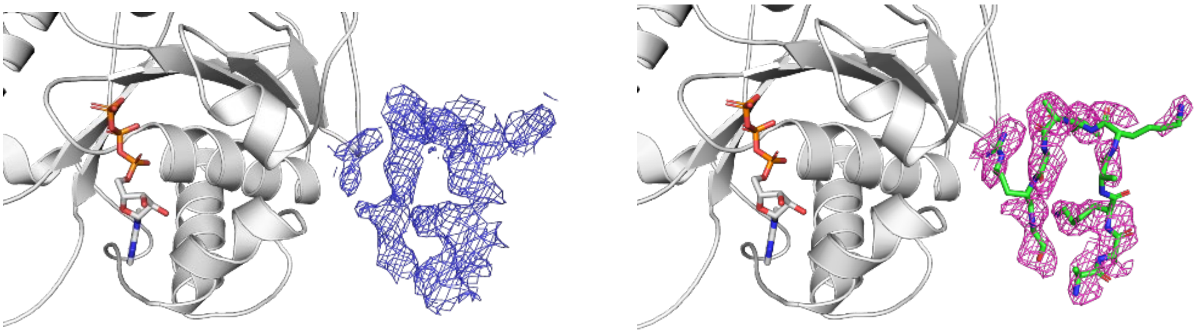

Supplementary Figure S4. The binding site of *Hp*ParAD41A-DNA-*Hp*ParBN10 complex. (A) The $2F_o$-F_c electron density map (blue mesh) contoured at 0.6 σ, calculated before addition of the *Hp*ParBN10 peptide to the *Hp*ParAD41A-DNA-*Hp*ParBN10 structure. (B) The $2F_o$-F_c electron density map (mesh in magenta) contoured at 0.6 σ, calculated after addition of the *Hp*ParBN10 peptide (sticks in green) to the *Hp*ParAD41A-DNA-*Hp*ParBN10 structure.

A B

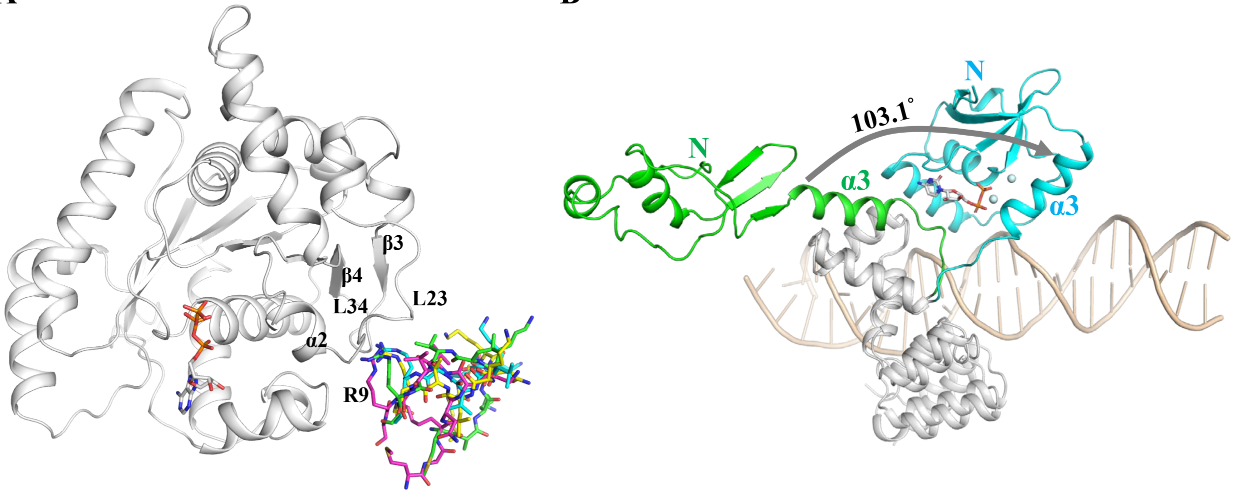

Supplementary Figure S5. Structural comparison of *Hp*ParB protomers and the NTD of *Hp*ParB and *Bs*ParB. (A) Superimposition of the four protomers from the asymmetric unit of the *Hp*ParAD41A-DNA-*Hp*ParBN10 complex. The four *Hp*ParBN10 peptides from each protomer are shown as sticks and colored in magenta, cyan, pink, and yellow, respectively. The one *Hp*ParAD41A monomer of the four protomers is shown and colored in white. (B) Superimposition of Ct-*Hp*ParB-*par*S complex (PDB ID: 4UMK) and *Bs*ParB-CDP-Ca^{2+} complex (PDB ID: 6SDK). The DBD of *Hp*ParB and *Bs*ParB monomer is fixed and colored in white. The NTD of *Hp*ParB and *Bs*ParB monomer is colored in green and cyan, respectively. The *N*-terminus and helix α4 of *Hp*ParB and *Bs*ParB are labeled as green and cyan, respectively. The CDP and two Ca^{2+} ions of *Bs*ParB are shown as sticks and balls.
